# Novel Conductometric Chemosensor Based on Methylenebis(Phosphonic Acid)-Modified Calixarene for L-Arginine Determination

**DOI:** 10.3390/mi17091047

**Published:** 2026-09-02

**Authors:** Svitlana V. Marchenko, Sergiy O. Cherenok, Kseniia O. Berketa, Veronika A. Bakhmat, Olha V. Soldatkina, Anna I. Selikhova, Olga I. Kalchenko, Galyna P. Volynets, Oleksandr O. Soldatkin, Vitaly I. Kalchenko, Sergei V. Dzyadevych, Abdelhamid Errachid, Viktoriya M. Pyeshkova

**Affiliations:** 1 Institute of Molecular Biology and Genetics, The National Academy of Sciences of Ukraine, 150 Zabolotnoho Str., 03143 Kyiv, Ukraine; 2 Institute of Organic Chemistry, The National Academy of Sciences of Ukraine, 5 Academician Kukhar Str., 02094 Kyiv, Ukraine; 3 National Technical University of Ukraine “Igor Sikorsky Kyiv Polytechnic Institute”, Department of Translational Medical Bioengineering, 37 Beresteysky Avenue, 03056 Kyiv, Ukraine; 4 Unité Mixte de Recherche 5280, Institut des Sciences Analytiques, Université Claude Bernard Lyon 1, Centre National de la Recherche Scientifique, 5 rue de la Doua, 69100 Villeurbanne, France; 5 School of Engineering, University of Warwick, Coventry CV4 7AL, UK

**Keywords:** L-arginine, chemosensor, conductometric transducer, calixarene, phosphonic acids, amino acids, supramolecular complexes

## Abstract

This study reports the synthesis of a novel methylenebisphosphonic acid calix[4]arene derivative and its application as a receptor layer in a conductometric chemosensor for the highly sensitive detection of L-arginine. The synthesized calixarene contains two methylenebisphosphonic acid fragments at the upper rim of the macrocycle, which serve as recognition sites for L-arginine, and two 3-(methylthio)propoxy groups at the lower rim, which enable immobilization on the gold electrode surface. The sensor based on synthesized calixarene demonstrated a pronounced response to L-arginine, which can be attributed to the cooperative interaction of the protonated amino and guanidinium groups of L-arginine with the methylenebisphosphonic acid groups of the calixarene receptor. Density functional theory (DFT) calculations were employed to elucidate the molecular interactions underlying the recognition of L-arginine by the calixarene receptor. The developed chemosensor demonstrated a low limit of detection for L-arginine (5.6 µM); a wide linear range (up to 1000 μM); a short response time (≤80 s); and a high response reproducibility (RSD = 1.3%). The stability constants determined by HPLC depended on the nature of the amino acid and ranged from logK_A_ = 4.26 for leucine to logK_A_ = 4.52 for L-arginine. Compared with previously reported L-arginine sensors, the developed conductometric chemosensor exhibits a competitive detection limit, a wider linear detection range, and significantly improved response reproducibility. The proposed calixarene-based chemosensor enables simple, highly sensitive, enzyme-free conductometric detection of L-arginine over a broad concentration range in aqueous solutions.

## 1. Introduction

L-arginine is a multifunctional amino acid involved in numerous metabolic pathways and serves as a precursor for several biologically active compounds, including nitric oxide, polyamines, creatine, and urea. It plays important roles in endothelial function [1,2], immune response [3,4], hormone secretion [5,6,7], and regulation of insulin sensitivity [8]. Consequently, the sensitive and selective detection of L-arginine is important for clinical diagnostics, biomedical research, nutritional analysis, food-quality monitoring, and quality control of pharmaceutical products. Among the various analytical approaches proposed for arginine detection, electrochemical sensors represent attractive alternatives to conventional chromatographic and spectroscopic methods owing to their simplicity, low cost, rapid response, and compatibility with miniaturized sensing platforms [9,10,11,12,13,14,15]. The performance of such devices is largely determined by the properties of the molecular recognition element responsible for selective binding of the target analyte. Therefore, the design of synthetic receptors capable of selective recognition of L-arginine remains an important task in arginine sensor development [16,17,18].

Calixarenes are widely studied synthetic macrocyclic receptors due to their well-defined three-dimensional structure, ease of synthesis, and versatile functionalization [19,20,21,22]. The cup-shaped calix[4]arene scaffold enables selective molecular recognition, while modification of its upper and lower rims allows the introduction of functional groups responsible for guest binding [23]. These features have supported broad applications in molecular recognition, chemical sensing, separation, and biomedicine [24,25,26].

Calixarenes, which are commonly used as synthetic receptors incorporated into chemosensors, offer important stability advantages over enzymes commonly used in biosensors. Unlike biological enzymes, they are not susceptible to denaturation, degradation, or loss of their structure when exposed to high temperatures, extreme pH levels, or organic solvents. Their high chemical and thermal stability can therefore support prolonged sensor operation, improved storage stability, and potential application in harsh analytical solutions [27].

Particularly attractive are calixarenes bearing phosphonic acid functionalities, which are capable of establishing multiple hydrogen-bonding and electrostatic interactions with charged and polar substrates [28,29,30]. The presence of several phosphonic acid groups within a single macrocyclic framework creates highly organized recognition sites suitable for binding amino acids, peptides, and proteins in aqueous media. At the same time, incorporation of sulfur-containing substituents at the lower rim enables efficient immobilization of calixarene receptors on gold surfaces through the formation of self-assembled monolayers. Such a combination of recognition and anchoring functions within a single molecule makes bifunctional calixarenes promising building blocks for the development of electrochemical sensor systems, in particular conductometric ones.

Previously, we proposed a bifunctional calix[4]arene receptor containing four methylenebisphosphonic acid fragments at the upper rim and two methylthiopropoxy groups at the lower rim. In this design, the methylenebisphosphonic acid groups were responsible for amino acid recognition, whereas the sulfur-containing substituents provided efficient immobilization on gold electrodes of conductometric transducers. The resulting receptor successfully formed supramolecular complexes with amino acids and served as an active sensing layer for conductometric detection. However, despite efficient host–guest interactions, the receptor exhibited limited selectivity toward individual amino acids [31].

We hypothesized that the presence of four methylenebisphosphonic acid fragments creates an extended and highly flexible interaction surface capable of establishing multiple binding modes with structurally different amino acids. Although such an arrangement favors strong binding, it may simultaneously reduce molecular discrimination and thus limit sensor selectivity. To overcome this limitation, a new receptor architecture was designed in which the number of methylenebisphosphonic acid fragments at the upper rim was reduced from four to two, while the methylthiopropoxy anchoring groups at the lower rim were retained. Such a modification was expected to generate a more geometrically defined binding site and thereby enhance selective recognition of L-arginine.

**Figure 1:**
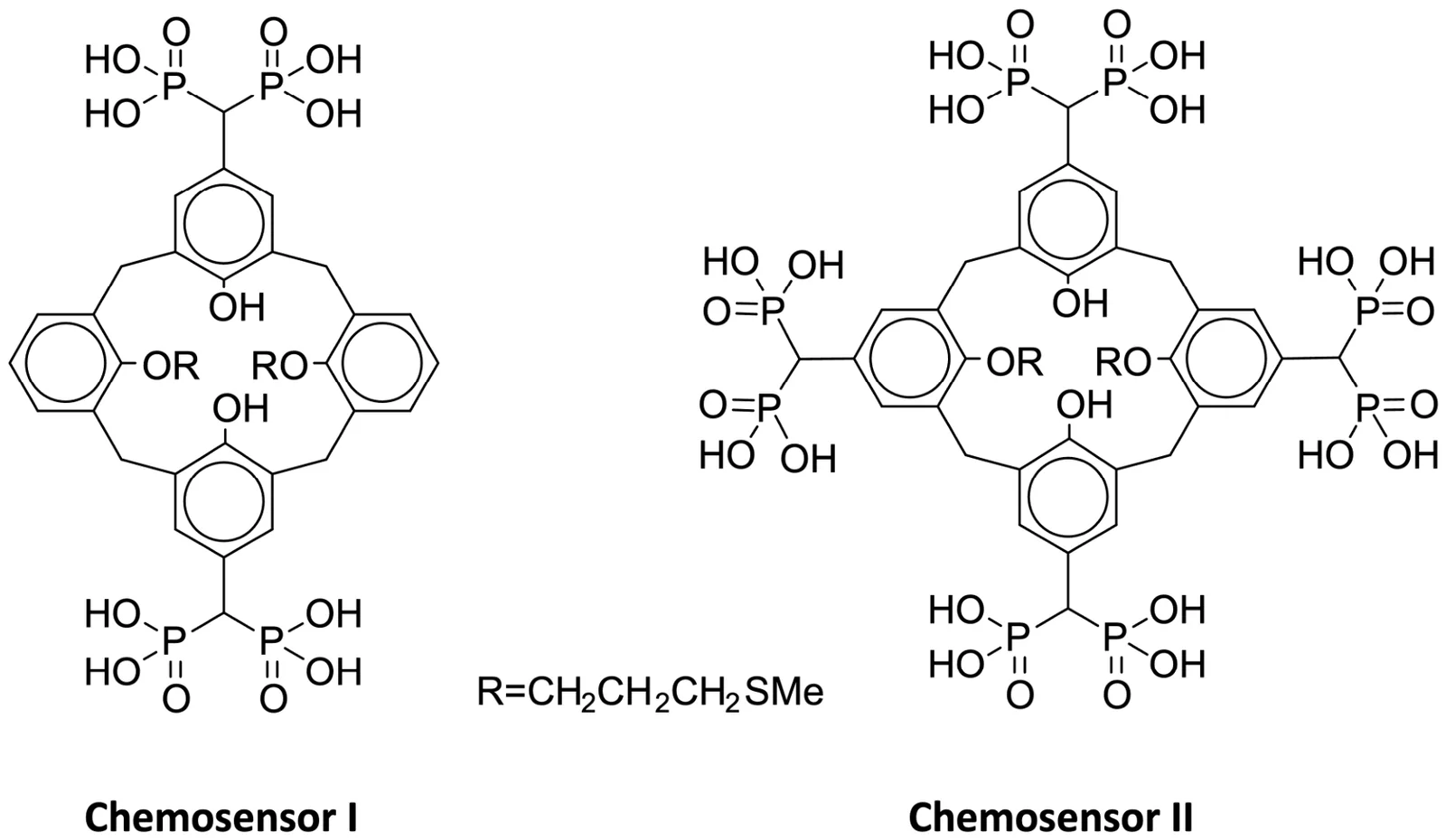
Structures of the calixarene receptors used in Chemosensors I and II.

Therefore, the aim of the present work was to synthesize and investigate a new bifunctional calix[4]arene receptor containing two methylenebisphosphonic acid fragments and two methylthiopropoxy groups as molecular recognition elements for the development of a conductometric chemosensor for L-arginine determination (Chemosensor I) (Figure 1). Particular attention was devoted to elucidating the structural factors governing L-arginine binding and selectivity. To achieve this goal, experimental studies were complemented by DFT calculations, which provided insight into the molecular recognition mechanism and revealed the important role of proton transfer, ion-pair formation, and cooperative hydrogen-bonding interactions in stabilization of the host–guest complex.

In this paper, we developed a new conductometric chemosensor (Chemosensor I), evaluated its main analytical characteristics, and compared its performance with that of a previously developed conductometric chemosensor (Chemosensor II) described in [31] (Figure 1).

## 2. Materials and Methods

### 2.1. Materials

L-amino acids were obtained from Sigma (St. Louis, MO, USA). L-arginine monohydrochloride, metallic sodium, diisopropyl phosphite, and 3-(methylthio)propanol were purchased from Sigma-Aldrich (Schnelldorf, Germany).

The starting formylcalixarene **1** was synthesized according to the reported procedure [32]. Dimethyl sulfoxide (DMSO) was used as a solvent for the preparation of calixarene solutions. KH_2_PO_4_ and Na_2_HPO_4_ for the potassium phosphate buffer solution were provided by “Chemlaborreaktiv” (Brovary, Ukraine) and had a purity grade of “p.a.” (pure for analysis) and “chem. pur.” (chemically pure). The solvents were dried according to standard procedures. All reactions were performed under an argon atmosphere using freshly distilled solvents. Organic solvents used in this work were obtained from UOSLAB (Kyiv, Ukraine). All other inorganic reagents were of analytical grade and used as received from commercial suppliers without further purification.

### 2.2. Synthesis of Calixarene Derivatives

#### 2.2.1. Synthesis of 5,17-Bis(bis(diisopropoxyphosphonyl)methyl)calix[4]arene **2**

Metallic sodium (10 mmol) was gradually dissolved in diisopropyl phosphite (5 mL) under cooling. After complete dissolution, the reaction mixture was degassed under reduced pressure (12 mmHg) for 5 min to remove dissolved hydrogen. Dry tetrahydrofuran (THF) (30 mL) and formylcalixarene **1** (2 mmol) were added to the resulting solution of sodium dialkyl phosphite. The reaction mixture was stirred at room temperature for 48 h. After completion of the reaction, the mixture was acidified to pH 6 with 1% aqueous HCl and diluted with water (100 mL). The product was extracted with chloroform (3 × 100 mL). The combined organic layers were concentrated under reduced pressure, and the residue was dried under vacuum (0.01 mmHg) at 50 °C for 10 h. The resulting oily residue was washed with hexane, and the precipitated white amorphous powder was collected by filtration and dried in a drying oven.

White amorphous powder: 92% yield, mp = 78–80 °C; ^1^H-NMR (CD_3_OD), δ: 0.58, 1.10,1.21, 1.34 (four d, 12H + 12H + 12H + 12H, *J* = 6.2 Hz, diastereotopic POCH(C***H***_3_)_2_), 3.67 (t, 2H, *J_PH_* = 25 Hz, P_2_C***H***), 3.5–4.2 (wide s, 8H, ArC***H***_2_), 4.27, 4.62 (two m, 4H + 4H, diastereotopic POC***H***(CH_3_)_2_), 6.65 (t, 2H, *J* = 7.5 Hz, *p*-Ar***H***), 7.08 (d, 4H, *J* = 7.5 Hz, *m*-Ar***H***), 7.27 (s, 4H, *m*-Ar***H***); ^31^P-NMR(CD_3_OD), *δ*: 17.00 (d, *J_PH_* = 25 Hz, ***P***_2_CH); ^13^C NMR (CD_3_OD), δ: 21.8, 22.9, 23.3 (three s), 30.7 (s, Ph***C***Ph), 44.90 (t, J_P-C_ = 146 Hz, P_2_-***C***), 71.7 and 72.7 (two s, O***C***H), 121.4 and 122.5 (two s), 128.1 (s), 128.5, 129.0 (two s), 131.2 (c), 148.2, 148.6 (two s, ***C***-OH). MS (HRMS) *m*/*z* 1108.4397 calcd for C_54_H_80_O_16_P_4_ [M]^+^, found 1109.4470 [M + H]^+^, 1131.4290 [M + Na]^+^. Anal. calcd for C_54_H_80_O_16_P_4_, %: C 58.48; H 7.27; P 11.17; found, %: C 58.42; H 7.36; P 10.99.

#### 2.2.2. Alkylation of Calixarenebisphosphonate **2** via the Mitsunobu Reaction

To a solution of tetrahydroxycalixarene bisphosphonate **2** (1 mmol) in dry THF (50 mL), triphenylphosphine (3 mmol) and 3-(methylthio)propanol (3 mmol) were added. Diethyl azodicarboxylate (3 mmol) was then added dropwise over 30 min with continuous stirring. The reaction mixture was stirred at room temperature for 5 h. The solvent was removed under reduced pressure (12 mmHg), and the crude product was purified by column chromatography using CHCl_3_/MeOH (10:1) as the eluent. As a result, a mixture of 5,17-di(bis(diisopropoxyphosphonyl)methyl)-25,27-bis(3-(methylthio)propoxy)calix[4]arene) **3.1** and 5,17-Di(bis(diisopropoxyphosphonyl)methyl)-26,28-bis(3-(methylthio)propoxy)calix[4]arene **3.2** was obtained.

##### 5,17-Di(bis(diisopropoxyphosphonyl)methyl)-25,27-bis(3-(methylthio)propoxy)calix[4]arene) **3.1**

White crystalline solid: 68% yield, mp = 68–70 °C; ^1^H-NMR (CDCl_3_), δ: 0.65 (wide s, 12H, diastereotopic POCH(C***H***_3_)_2_), 1.20–1.31 (m, 24H + 12H, diastereotopic POCH(C***H***_3_)_2_,), 2.17 (s, 6H, SC***H***_3_), 2.26 (m, 4H, C***H***_2_), 3.01 (m, 4H, C***H***_2_), 3.32 (d, 4H, *J* = 13.2 Hz, ArC***H***_2eq_), 3.48 (t, 2H, *J_PH_* = 25 Hz, P_2_C***H***), 4.08 (t, 4H, J = 5.9 Hz, OC***H***_2_), 4.23 (d, 4H, ArC***H***_2*ax*_*, J* = 13.2 Hz), 4.48, 4.68 (two m, 4H + 4H, diastereotopic POC***H***(CH_3_)_2_), 6.54 (t, 2H, *J* = 7.5 Hz, *p*-Ar***H***), 6.83 (d, 4H, *J* = 7.5 Hz, *m*-Ar***H***), 7.18 (wide s, 4H, *m*-Ar***H***), 7.82 (s, 4H, PhO***H***); ^31^P-NMR (CDCl_3_), δ: 19.4; ^13^C NMR (CDCl_3_), 17.1, 21.8, 22.9, 23.3, 29.7 (s, Ph***C***Ph), 30.7, 30.73, 44.3 (t, J_P-C_ = 145 Hz, P_2_-***C***), 69.7, 71.2, 75.1, 121.2, 125.3, 128.3, 130.1, 131.9, 133.7, 152.1, 153.6; MS (HRMS) *m/z* 1284.5090 calcd for C_62_H_96_O_16_P_4_S_2_ [M]^+^, found 1285.5169 [M + H]^+^, 1307.4986 [M + Na]^+^. Anal. calcd for C_62_H_96_O_16_P_4_S_2_, %: C 57.93; H 7.53; P 9.64; found, %: C 57.85; H 8.02; P 9.56.

##### 5,17-Di(bis(diisopropoxyphosphonyl)methyl)-26,28-bis(3-(methylthio)propoxy)calix[4]arene **3.2**

White crystalline solid: 11% yield, mp = 64–66 °C; ^1^H-NMR (CDCl_3_), δ: 0.47 (d, 12H, *J* = 5.7 Hz, diastereotopic POCH(C***H***_3_)_2_), 1.1–1.19 (m, 24H + 12H, diastereotopic POCH(C***H***_3_)_2_), 2.18 (s, 6 H, SC***H***_3_), 2.30 (m, 4 H, C***H***_2_), 3.04 (m, 4H, C***H***_2_), 3.31 (t, 2H, *J_PH_* = 25 Hz, P_2_C***H***), 3.37 (d, 4H, *J* = 13.2 Hz, ArC***H***_2eq_), 4.06 (t, 4H, J = 5.9 Hz, OC***H***_2_), 4.27 (d, 4H, ArC***H***_2*ax*_*, J* = 13.2 Hz), 4.18, 4.59 (two m, 4H + 4H, diastereotopic POC***H***(CH_3_)_2_), 6.60 (t, 2H, *J* = 7.5 Hz, *p*-Ar***H***), 7.01 (d, 4H, *J* = 7.5 Hz, *m*-Ar***H***), 7.10 (s, 4H, *m*-Ar***H***), 8.36 (s, 4H, PhO***H***); ^31^P-NMR (CDCl_3_), δ: 19.1; ^13^C NMR (CDCl_3_), 15.1, 22.3, 22.7, 22.9, 28.3, 31.5, 32.73, 45.2 (t, J_P-C_ = 145 Hz, P_2_-***C***), 70.5, 72.3, 74.1, 118.4, 125.1, 127.6, 127.9, 128.3, 133.1, 151.4, 152.7; MS (ESI) *m/z* 1284.5090 calcd for C_62_H_96_O_16_P_4_S_2_ [M]^+^, found 1285.5170 [M + H]^+^, 1307.4983 [M + Na]^+^.

#### 2.2.3. Synthesis of 5,17-Di(bis(dihydroxyphosphoryl)methyl)-25,27-bis(3-(methylthio)propoxy)calix[4]arene **4** (Chemosensor I Receptor)

To a solution of calixarene phosphonate **3.1** (1 mmol) in dry chloroform (5 mL), bromotrimethylsilane (5 equiv per phosphoryl group) was added. The reaction mixture was stirred at room temperature for 72 h. The solvent was removed under reduced pressure, and the residue was dissolved in methanol (15 mL). The resulting solution was stirred at 40 °C for 4 h and then concentrated under reduced pressure. The residue was dried under vacuum (0.05 mmHg) for 10 h.

A pale-colored amorphous solid: 94% yield, mp = 121–125 °C; ^1^H-NMR (CD_3_OD), δ: 2.20 (s, 6H, SC***H***_3_), 2.32 (m, 4H, C***H***_2_), 3.13 (m, 4H, C***H***_2_), 3.40 (d, 4H, *J* = 13.2 Hz, ArC***H***_2eq_), 3.52 (t, 2H, *J_PH_* = 25 Hz, P_2_C***H***), 4.13 (t, 4H, J = 5.9 Hz, OC***H***_2_), 4.33 (d, 4H, *J* = 13.2 Hz, ArC***H***_2*ax*_), 6.74 (t, 2H, *J* = 7.5 Hz, *p*-Ar***H***), 7.05 (d, 4H, *J* = 7.5 Hz, *m*-Ar***H***), 7.28 (s, 4H, *m*-Ar***H***); ^31^P-NMR (CD_3_OD), δ: 17.5; ^13^C NMR (CD_3_OD), 14.3, 30.2, 31.4, 32.9, 45.2 (t, J_P-C_ = 144.3 Hz, P_2_-***C***), 74.6, 122.3, 127.1, 127.14, 127.9, 128.3, 128.4, 130.3, 147.8, 148.4); MS (HRMS) *m/z* 948.1334 calcd for C_38_H_48_O_16_P_4_S_2_ [M]^+^, found 949.1413 [M + H]^+^, 1897.2741 [2M + H]^+^. Anal. calcd for C_38_H_48_O_16_P_4_S_2_, %: C 48.10; H 5.10; P 13.06; found, %: C 48.17; H 5.02; P 13.14.

### 2.3. Reverse-Phase High-Performance Liquid Chromatography (RP HPLC) Analysis

RP HPLC analyses were performed using a Hitachi chromatography system in a mobile phase of water/acetonitrile/methanol (80/15/5 by volume) on a Zorbax CN column (250 × 4.6 mm). The ultraviolet detector was set to a wavelength of 254 nm for calixarene analysis and 210 nm for amino acid analysis. The mobile-phase flow rate was 0.6 mL/min. The experiment was performed under isocratic conditions. Chromatograms were obtained at 26 °C. The mobile phase contained Chemosensor I receptor additives at concentrations of 0.02–0.1 mM. Amino acid samples for injection were dissolved in a mixture of water/acetonitrile/methanol (80/15/5 by volume) (concentration 0.01 mM). The volume of the injected samples was 20 μL. Before analysis, the column was stabilized for 3 h with a mobile phase containing the calixarene additive.

### 2.4. Density Functional Theory Calculations

DFT calculations were carried out using the ORCA 6.1.0 program package [33]. Geometry optimizations of the free receptor and its complex with L-arginine were performed employing the ωB97X-D3 exchange-correlation functional in conjunction with the def2-SVP basis set. The RIJCOSX approximation was applied to accelerate the calculations. Solvent effects were taken into account using the CPCM (conductor-like polarizable continuum model) combined with the SMD (solvation model based on density) for water. All systems were calculated as neutral singlets. For the amino acid complexes, the optimized structures correspond to proton-transfer ion-pair forms, in which the receptor is partially deprotonated and the amino acid fragments are protonated at the α-amino group.

Frequency calculations were performed for all optimized structures at the same level of theory. The absence of imaginary frequencies confirmed that the obtained geometries correspond to local minima on the potential energy surface.

To reduce the computational cost, the two 3-(methylthio)propoxy substituents located at the lower rim of the calix[4]arene receptor were replaced by propoxy groups. As these substituents are spatially separated from the binding site and do not participate directly in complex formation, such simplification is not expected to affect the calculated binding mode or the hydrogen-bonding pattern within the complex.

Molecular structures and graphical representations were generated in ChimeraX software packages (version 1.11) [34].

The Cartesian coordinates of the optimized structures of Chemosensors I and II receptor–L-arginine complexes are provided in the Supplementary Material.

### 2.5. Conductometric Sensor Design

**Figure 2:**
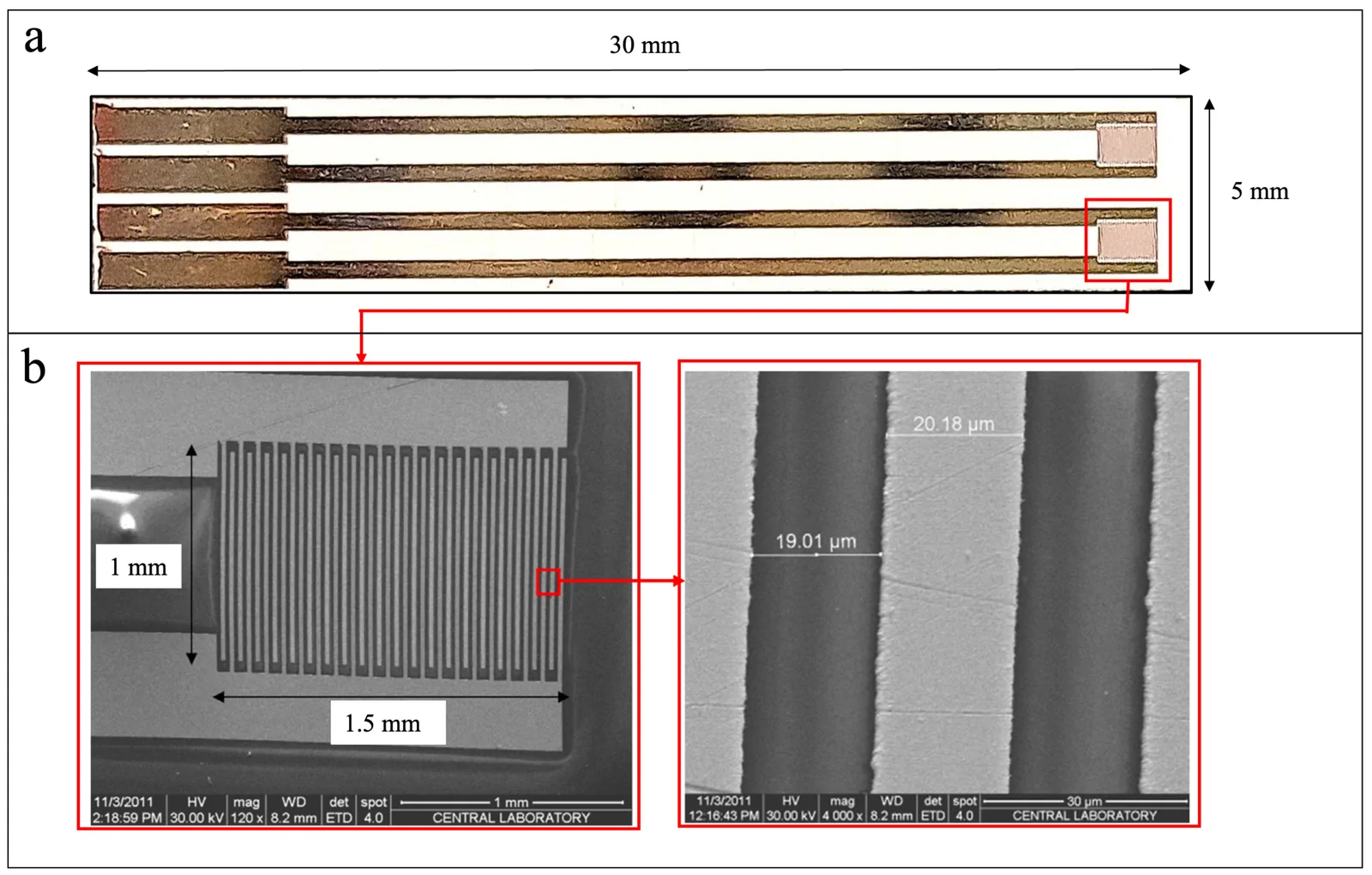
(**a**) Photograph of the conductometric transducer; (**b**) image of gold interdigitated electrodes obtained by scanning electron microscopy.

Planar thin-film conductometric transducers were used for calixarene immobilization. Each transducer consisted of two identical pairs of gold interdigitated electrodes. The transducers were fabricated at the V. Ye. Lashkaryov Institute of Semiconductor Physics of the National Academy of Sciences of Ukraine according to the authors’ specifications. Gold electrodes were prepared by vapor deposition onto a non-conductive pyroceramic substrate (5 × 30 mm). To improve adhesion of the gold layer to the substrate, an intermediate chromium layer (5 nm) was deposited prior to application of the gold layer (150 nm). Each electrode pair possessed an interdigitated comb-like geometry with a digit width and interdigital spacing of 20 μm, while the digit length was approximately 1.5 mm. The conductive buses were coated with an insulating material, leaving only the active sensing area and the contact region exposed for sensor fixation in the electrode holder. Figure 2 illustrates the conductometric transducer employed in the present study.

### 2.6. Immobilization of Calixarene onto Conductometric Transducers

Prior to deposition of the sensor selective layers, the surface of the conductometric transducers was cleaned using a freshly prepared Piranha solution. The electrodes were immersed in the cleaning solution at 60–80 °C for 10–15 min, followed by thorough rinsing with several portions of double-distilled water. Subsequently, the transducers were treated with ethanol and dried completely before further use.

The sulfur-containing groups of the synthesized Chemosensor I receptor facilitated its adsorption onto the gold surface of the conductometric transducers.

The calixarene sensing layer was deposited onto the transducer surface by a drop-casting procedure. Calixarene solutions were prepared in DMSO. A fixed volume (0.1 μL) of the calixarene solution was applied onto the active area of each pair of gold electrodes. Subsequently, the modified transducers were heated at 80 °C for 5 min to promote formation of the sensing layer. This treatment did not affect the operational characteristics of the transducers. As a result, a stable calixarene film was formed within the active electrode regions.

### 2.7. Measurement Equipment and Analytical Procedure

**Figure 3:**
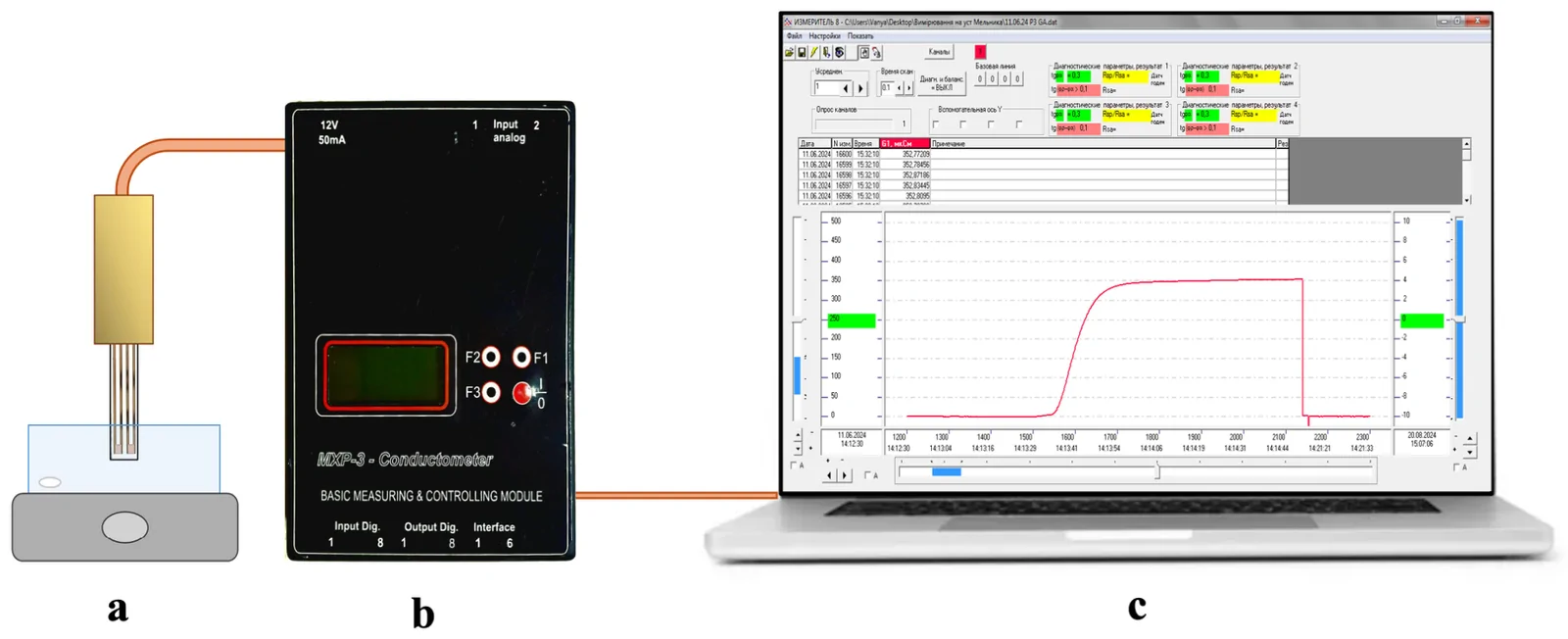
Conductometric measuring system. (**a**) Electrodes immersed in the buffer solution in a cell placed on a magnetic stirrer; (**b**) portable conductometer MXP-3; (**c**) personal computer with specialized software for MXP-3 operation and data acquisition.

Chemosensor measurements were performed in a differential mode using a conductometric analyzer developed in collaboration with the Institute of Electrodynamics of the National Academy of Sciences of Ukraine. The analyzer enabled detection of local changes in solution conductivity according to the conductometric measurement principle. The bridge measuring circuit of the conductometric analyzer operated at a supply voltage of 14 mV with an applied frequency of 66 kHz. The experimental setup consisted of an electronic measuring module, a sensor holder, a measuring cell containing the working buffer solution, a magnetic stirrer, and the appropriate software for data acquisition and processing (Figure 3).

The operational characteristics of the conductometric analyzer minimized undesirable electrochemical effects, including Faradaic processes, double-layer charging, and electrode polarization. In addition, a differential measurement mode was employed to suppress non-informative environmental influences, such as local variations in temperature, pH, and background conductivity of the working buffer solution.

All measurements were conducted at room temperature in an open electrochemical cell (2.0 mL) containing the working buffer solution under continuous stirring. The concentration of L-arginine in the working cell was set by adding the initial stock solution. Each sample was analyzed in 3–5 replicates.

## 3. Results and Discussion

### 3.1. Chemical Synthesis of the Chemosensor I Receptor

**Figure 4:**
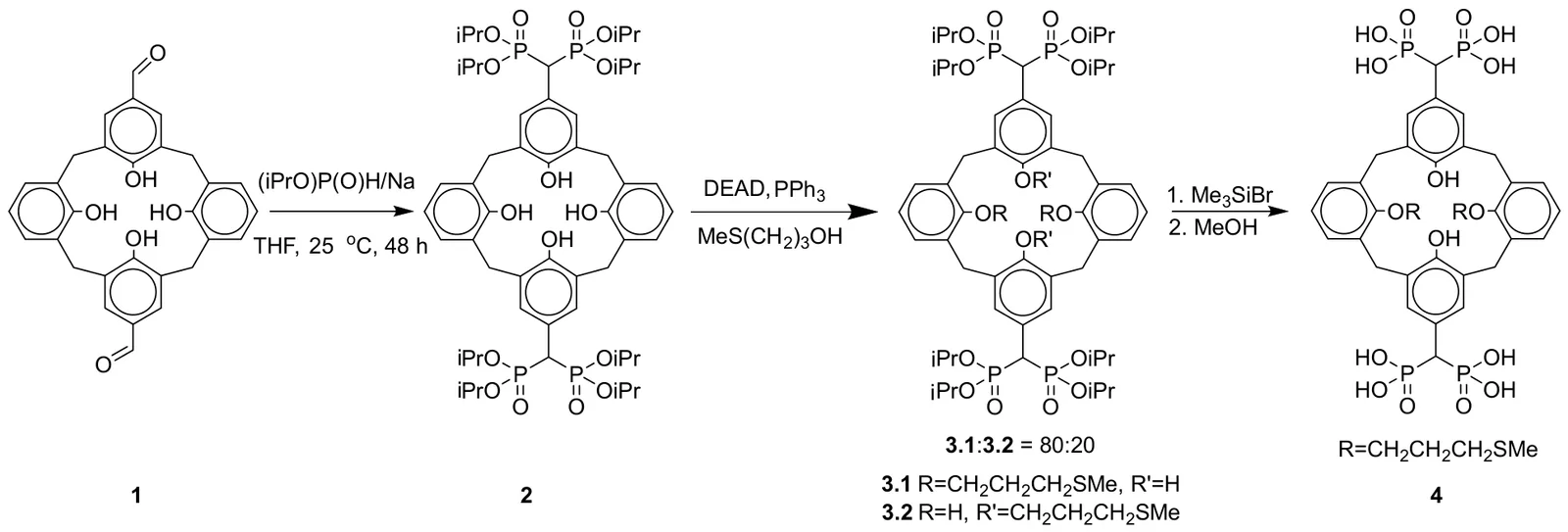
Synthesis of di(3-methylthiopropoxy)calixarene bis(methylenebisphosphonic acid) **4**, the Chemosensor I receptor.

The synthesis of calixarene bisphosphonic acid (the Chemosensor I receptor) bearing two methylthiopropoxy fragments was accomplished in several steps (Figure 4). In the first stage, diformyltetrahydroxycalixarene **1** was treated with an excess of diisopropyl phosphite in the presence of a tenfold excess of metallic sodium to afford calixarene methylenebisphosphonate **2** (Figures S1 and S2) [35,36].

The Mitsunobu reaction was used for the introduction of two 3-(methylthio)propoxy groups onto the lower rim of calixarene methylenebisphosphonic acid diisopropyl ester **2**. The reaction with 3-(methylthio)propanol was carried out in anhydrous THF at 20–25 °C in the presence of triphenylphosphine and diethyl azodicarboxylate (DEAD) and was complete within 4–5 h. At a calixarene/PPh_3_/DEAD/alcohol molar ratio of 1:3:3:3, a mixture of distally dialkylated calixarenes **3.1** and **3.2** was obtained in an 80:20 ratio (Figure 4). Increasing the amount of alcohol, reaction temperature, or reaction time did not result in the formation of tri- or tetraalkylated products.

The isolated calixarene methylenebisphosphonates **3.1** and **3.2** were found to exist in a *flattened-cone* conformation. Evidence for this conformation comes from the ^1^H NMR (Nuclear Magnetic Resonance) spectra, which exhibit two doublets of an AB spin system assigned to the axial and equatorial protons of the ArCH_2_Ar methylene bridges. The chemical shift difference between these signals (Δδ = 0.89–0.90 ppm) and the geminal coupling constant (J_HH_ = 13 Hz) are characteristic of the *flattened-cone* conformation [37].

The spatial structures of calixarenes **3.1** and **3.2** were confirmed by 2D HMBC (Heteronuclear Multiple-Bond Correlation) NMR spectroscopy, which enables the detection of correlations between carbon and proton nuclei separated by two, three, or, in conjugated systems, four bonds (Figures S3 and S5).

In the HMBC spectrum of bisphosphonate **3.1**, a cross-peak was observed between the triplet signal of the OCH_2_ protons (δ 4.08 ppm) and the carbon signal at δ 151.79 ppm assigned to the *para*-unsubstituted phenolic ring. In addition, a cross-peak was detected between the hydroxyl proton signal (δ 7.83 ppm) and the triplet carbon signal at δ 153.15 ppm corresponding to the para-substituted phenolic ring (Figure S4). These correlations unequivocally demonstrate that alkylation of calixarene **2** occurs predominantly at the hydroxyl groups of the *para*-unsubstituted phenolic units, leading to the formation of calixarene **3.1** as the major product. The regioisomeric calixarene **3.2** is formed as a minor alkylation product.

The Chemosensor I receptor was synthesized in nearly quantitative yield via dealkylation of calixarene methylenebisphosphonate **3.1** with bromotrimethylsilane in chloroform (72 h at 20 °C), followed by methanolysis of the intermediate silyl esters with excess methanol (4 h at 40 °C) (Figure 4). The ^1^H NMR spectra of the Chemosensor I receptor revealed an AB spin system for the nonequivalent axial and equatorial protons of the ArCH_2_Ar methylene bridges (Δδ = 0.9 ppm), indicating that the molecule retains a flattened-cone conformation in solution (Figures S6 and S7).

**Figure 5:**
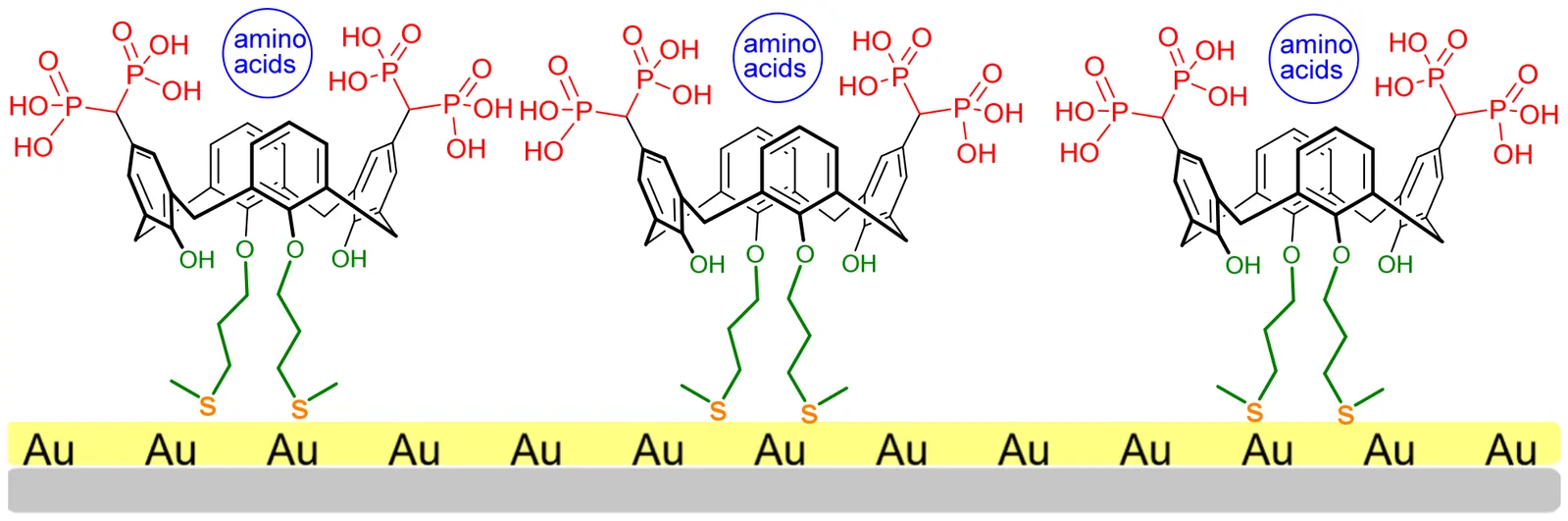
Schematic representation of a gold conductometric sensor surface coated with a self-assembled monolayer of the Chemosensor I receptor.

Thus, we synthesized a Chemosensor I receptor bearing two methylenebisphosphonic acid groups on the upper rim of the macrocycle. These groups are capable of forming host–guest supramolecular complexes with amino acids. At the same time, the Chemosensor I receptor contains two 3-(methylthio)propoxy substituents on the lower rim of the macrocycle. The sulfur-containing substituents provide efficient anchoring of calixarene onto the gold surface, making this compound a promising receptor layer for conductometric transducers designed for L-arginine detection. (Figure 5).

### 3.2. DFT Analysis of the Calixarene Receptor Complexes with L-Arginine

To gain deeper insight into the molecular basis of L-arginine recognition by the calixarene-based chemosensors, the structures of the calixarene–L-arginine complexes were investigated using density functional theory (DFT) calculations. The calculations were performed at the ωB97X-D3/def2-SVP level with the SMD solvation model for water. The optimized structures of both complexes were confirmed to correspond to minima on the potential energy surface by the absence of imaginary vibrational frequencies.

In the computational model, the two 3-(methylthio)propoxy substituents at the lower rim of the Chemosensor I and Chemosensor II receptors were replaced by propoxy groups to reduce the computational cost. These substituents are responsible for immobilization on the gold surface, but they are spatially separated from the upper-rim bisphosphonic acid binding site and are not directly involved in host–guest interactions. Therefore, this simplification is not expected to significantly affect either the preferred receptor conformation or the calculated binding modes of L-arginine.

**Figure 6:**
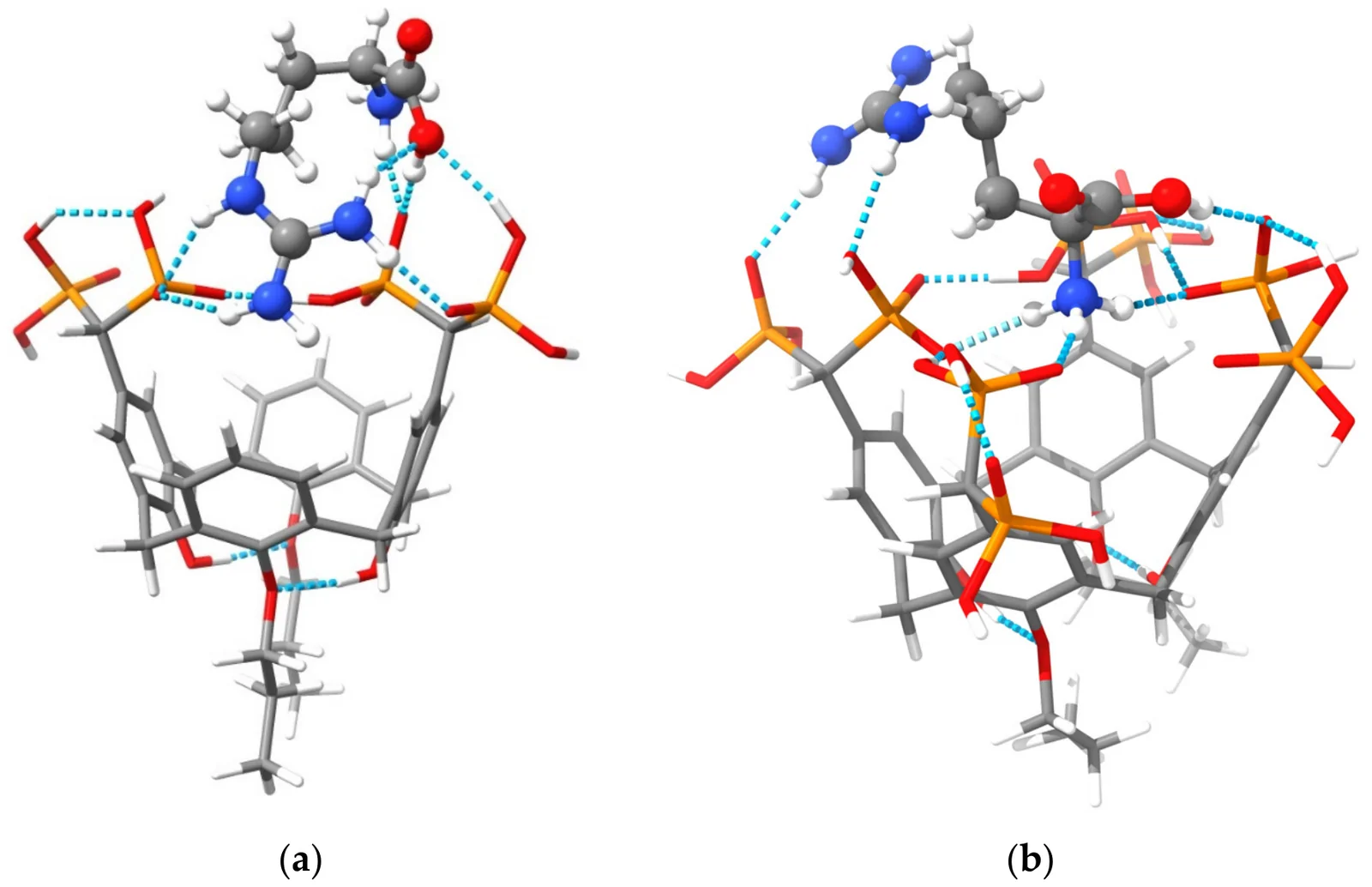
DFT-optimized structures of the L-arginine complexes with the Chemosensor I receptor (**a**) and Chemosensor II receptor (**b**). The 3-(methylthio)propoxy groups were replaced with propoxy groups in the computational models.

The optimized structures of the Chemosensor I and Chemosensor II receptor complexes with L-arginine adopt a *flattened-cone* conformation, which is stabilized by intramolecular hydrogen bonds at the lower rim of the macrocycle (Figure 6). These interactions restrict the conformational mobility of the calixarene framework and contribute to its preorganization.

The optimized structure of the Chemosensor I receptor–L-arginine complex reveals a well-defined host–guest arrangement stabilized by six intermolecular hydrogen bonds involving the guanidinium, protonated amino, and carboxyl groups of L-arginine and the phosphonate groups of the receptor (Table 1).

The most prominent structural feature is the spatial arrangement of the positively charged guanidinium group. It is positioned between the two distal methylenebisphosphonic acid groups, allowing the guanidinium functionality to interact with phosphonate oxygen atoms located on different sides of the binding region. This arrangement provides pronounced geometric and electrostatic complementarity between the calixarene receptor and the guanidinium group and contributes to the well-defined orientation of L-arginine within the binding cavity. The guanidinium group forms three N–H⋯O hydrogen bonds with phosphonate oxygen atoms. These interactions are characterized by H⋯O distances of 1.77–2.03 Å and N–H⋯O angles of 147.4–166.8°, indicating favorable and directional hydrogen-bonding interactions. The protonated amino group forms an additional N–H⋯O hydrogen bond with a phosphonate oxygen atom, with an H⋯O distance of 1.64 Å and an N–H⋯O angle of 157.2°.

A particularly strong interaction is observed between the carboxyl group of L-arginine and a phosphonate oxygen atom of the receptor, with an H⋯O distance of 1.44 Å, a D⋯A distance of 2.47 Å, and an O–H⋯O angle of 175.1°. The remaining hydrogen bonds further contribute to the stabilization of the host–guest assembly. Collectively, these interactions establish a highly organized hydrogen-bonding network that fixes the orientation of L-arginine relative to the phosphonate-containing upper rim of the Chemosensor I receptor.

The optimized structure of the Chemosensor II receptor–L-arginine complex exhibits a more extensively functionalized hydrogen-bonding surface owing to the presence of four methylenebisphosphonic acid groups. As in the Chemosensor I receptor complex, six intermolecular hydrogen bonds are identified between L-arginine and the phosphonate groups of the receptor (Table 1). Three of these interactions are formed by the protonated amino group of arginine, which is partially embedded within the calixarene cavity and acts as a three-point hydrogen-bond donor toward oxygen atoms of neighboring methylenebisphosphonic acid groups. These interactions are characterized by H⋯O distances of 1.67–1.97 Å and N–H⋯O angles of 154.3–168.5°, respectively, indicating a set of short and directional contacts that collectively anchor the protonated amino group within the phosphorylated calixarene cavity.

The protonated guanidinium group is located above the upper-rim phosphonate network rather than deeply within the calixarene cavity. It forms two additional N–H⋯O hydrogen bonds with phosphonate oxygen atoms, characterized by H⋯O distances of 1.94–2.00 Å and N–H⋯O angles of 154.0–166.8°. The sixth intermolecular interaction involves the carboxyl group of L-arginine and a phosphonate oxygen atom. This O–H⋯O hydrogen bond has an H⋯O distance of 1.63 Å and an O–H⋯O angle of 163.1°.

Thus, the Chemosensor II receptor provides a more densely functionalized binding surface in which the available phosphonate groups can establish multiple directional contacts with the different hydrogen-bond donor sites of L-arginine. Compared with the Chemosensor I receptor, the additional methylenebisphosphonic acid groups increase the overall number of accessible interaction sites without substantially changing the ability of the calixarene framework to accommodate the amino acid.

To quantitatively compare the stabilization of the L-arginine complexes with the Chemosensor I and II receptors, the corresponding DFT interaction energies were calculated from the total electronic energies of the optimized host–guest complexes and the isolated receptors and L-arginine. The calculated interaction energy, ΔE_int_, was −244.8 kJ mol^−1^ (−58.5 kcal mol^−1^) for the Chemosensor I receptor–L-arginine complex and −269.2 kJ mol^−1^ (−64.4 kcal mol^−1^) for the Chemosensor II receptor–L-arginine complex. Thus, the interaction with the Chemosensor II receptor is more favorable by approximately 24.4 kJ mol^−1^ (5.8 kcal mol^−1^).

The relatively large absolute values of the calculated interaction energies should be interpreted with caution. ΔE_int_ represents the difference between the total electronic energies of the optimized complex and the corresponding isolated components and therefore includes the cumulative effects of hydrogen bonding, electrostatic interactions, polarization, charge redistribution, and dispersion. These values should consequently be regarded primarily as internally consistent descriptors of host–guest stabilization rather than as absolute Gibbs free energies of binding or direct estimates of association constants. Accordingly, the difference between the two systems is more informative than the absolute magnitude of the calculated energies.

The more favorable interaction energy calculated for the Chemosensor II receptor is consistent with its larger number of methylenebisphosphonic acid groups and the resulting increase in the number of accessible phosphonate oxygen atoms. However, the stronger overall interaction does not necessarily imply greater molecular recognition selectivity. The additional phosphonate groups increase the interaction capacity of the Chemosensor II receptor, but some of the available hydrogen-bonding sites remain accessible for interactions with other amino-acid guests and may therefore contribute to less specific stabilization.

In contrast, the Chemosensor I receptor possesses a less extensively functionalized binding surface but provides a more spatially defined recognition environment. In particular, the positioning of the guanidinium group between the two distal methylenebisphosphonic acid groups creates a complementary arrangement of electrostatic and hydrogen-bonding interactions that may be particularly favorable for recognition of L-arginine.

Overall, the DFT results demonstrate that both calixarene receptors form well-defined complexes with L-arginine through multiple directional hydrogen bonds, while differing in the organization of their binding sites. The Chemosensor II receptor provides stronger calculated overall host–guest stabilization, whereas the Chemosensor I receptor generates a more spatially constrained recognition pattern centered on the guanidinium group. These results indicate that the molecular recognition properties of the calixarene receptors are governed not only by the number and strength of intermolecular interactions, but also by their spatial organization and geometric and electrostatic complementarity to the guest molecule.

### 3.3. Determination of Stability Constants of the Complexes of the Chemosensor I Receptor with Amino Acids

To understand the nature of molecular recognition and binding of amino acids by the developed sensor, the complexation of the Chemosensor I receptor with amino acids in the mobile phase was investigated by RP HPLC and molecular calculations. The Chemosensor I receptor in the flow of the mobile-phase water/acetonitrile/methanol (80/15/5 by volume) on the Zorbax CN column is recorded as a symmetrical peak with a retention time t_R_ = 6.958 min. Under these analysis conditions, all the studied amino acids are recorded as peaks with t_R_ values in the range of 4.30–28.0 min. The addition of the Chemosensor I receptor to the mobile phase leads to the formation of supramolecular complexes and is accompanied by a decrease in the values of the chromatographic parameters t_R_ and k’ of the amino acids (Figure S8). The linear dependences of 1/k′ of the amino acids on the concentration of the Chemosensor I receptor (Figure S9) indicate a 1:1 stoichiometry of the complexes and allow us to use Equation (1) [38,39] for the calculations of the K_A_.

1/k′ = 1/k_0_′ + K_A_ × [CA]/k_0_′ (1)

where k_0_′ and k′ are the amino acid capacity coefficients determined in the absence and presence of calixarene in the mobile phase.

The stability constants logK_A_, the Gibbs free energies ΔG (ΔG = -RTlnK_A_) of the complexes, and the L-arginine selectivity S, calculated as the ratio of the stability constants of the L-arginine complex to the complex of the reference amino acid AA (S = K_A_(Arg)/K_A_(AA)), are presented in Table 2.

The experimentally determined stability constants of the K_A_ complexes are in the range from logK_A_ = 4.260 to logK_A_ = 4.523, with the highest value observed for L-arginine. However, the L-arginine selectivity coefficients calculated vary only from 1.35 to 1.83, indicating only moderate thermodynamic preference for L-arginine under the RP-HPLC conditions.

In contrast, the conductometric response of the receptor immobilized on the gold electrode surface shows a much more pronounced selectivity toward L-arginine. This difference can be attributed to the fact that HPLC and conductometric measurements characterize different processes. The RP HPLC-derived (K_A_) values reflect the stability of host–guest complexes in bulk solution, whereas the sensor response depends not only on binding affinity, but also on the orientation and accessibility of the receptor in the monolayer, proton-transfer processes, charge distribution of the complexes, and changes in the interfacial ionic environment near the electrode surface.

Thus, the RP-HPLC and conductometric data should be considered complementary rather than directly proportional. The solution studies confirm the intrinsic ability of the Chemosensor I receptor to bind amino acids, while the surface measurements demonstrate how complex formation is translated into an analytical signal.

### 3.4. Calibration Curves and Analytical Characteristics of the Arginine-Sensitive Chemosensors

The analytical characteristics of Chemosensor I, developed in the present study, were compared with those of Chemosensor II, which was previously reported by our group [31].

**Figure 7:**
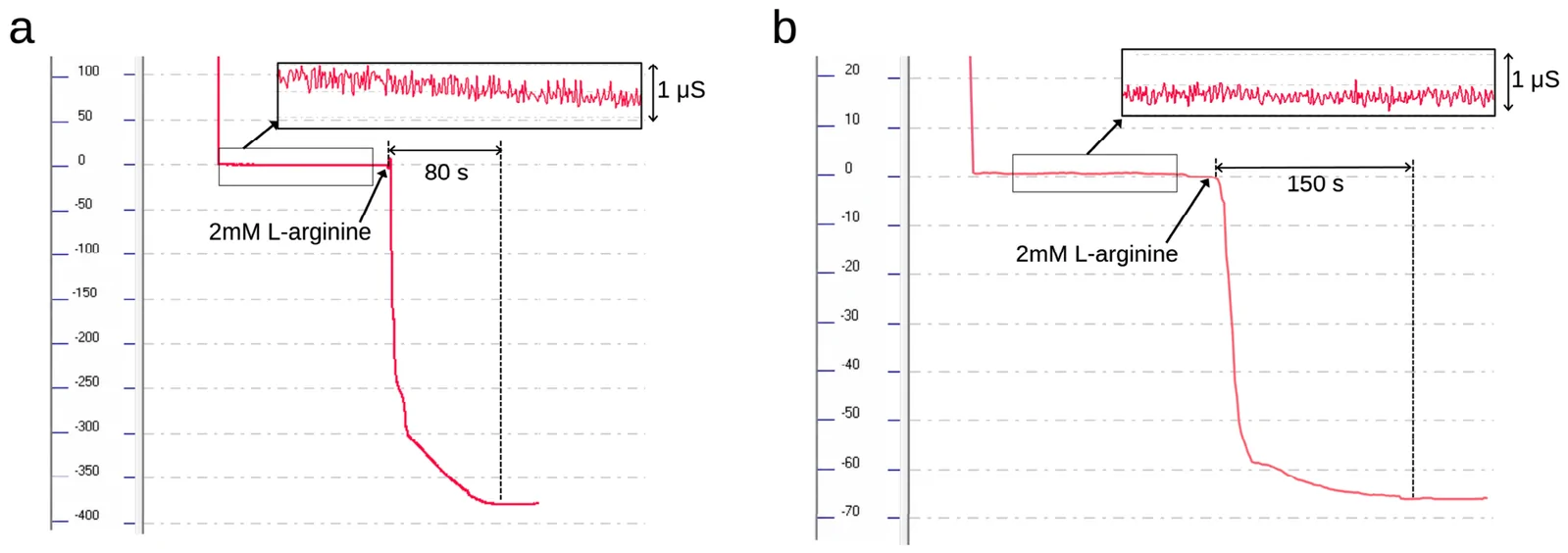
Typical responses of Chemosensor I (**a**) and Chemosensor II (**b**) following the addition of 2 mM L-arginine. Measurements were carried out in 5 mM phosphate buffer solution, pH 7.5.

**Figure 8:**
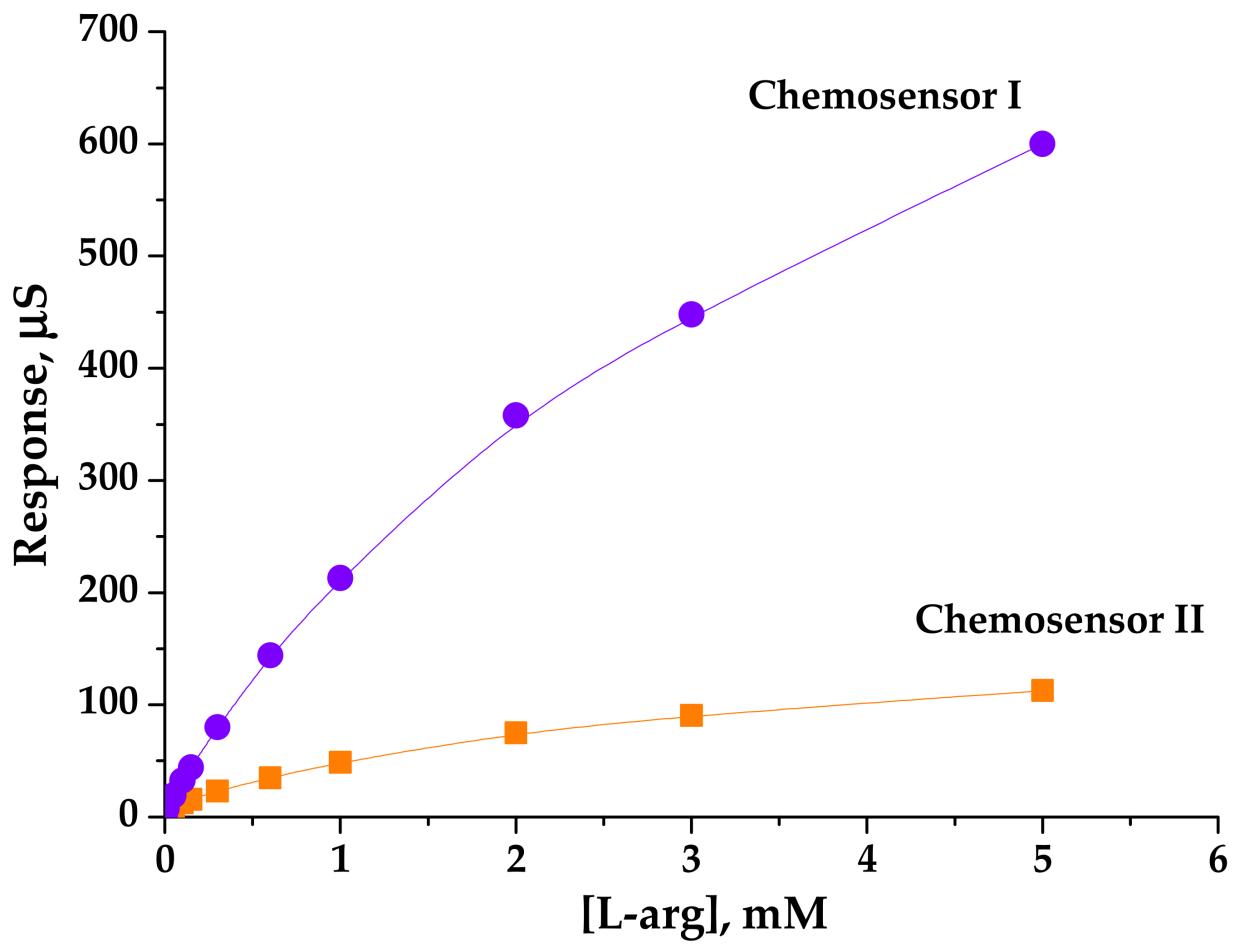
Calibration curves of Chemosensor I and Chemosensor II for L-arginine detection. Measurements were carried out in 5 mM phosphate buffer solution, pH 7.5.

First, the typical response curves (Figure 7 and Figure S10) and calibration curves (Figure 8) of the two types of conductometric chemosensors for L-arginine detection were compared.

As shown in Figure 7, the response profiles of both conductometric chemosensors were nearly identical in shape. The response time of Chemosensor I was almost twofold lower than that of Chemosensor II. The baseline noise was slightly higher for Chemosensor I, differing by only 0.125 μS from that observed for Chemosensor II.

Sensitivity and linear detection range are important performance characteristics of sensors intended for analyte determination in real samples. Therefore, we compared the calibration graphs of both chemosensors constructed using two different types of calixarenes. For this purpose, L-arginine solutions with concentrations ranging from 0.005 to 5 mM were used in the measurements. As can be seen from the calibration curves (Figure 8), the sensitivity of Chemosensor I toward 1 mM L-arginine was approximately four times higher than that of Chemosensor II.

In addition, the linear detection range of Chemosensor I for L-arginine was more than six times broader than that of Chemosensor II (Figure 8).

**Figure 9:**
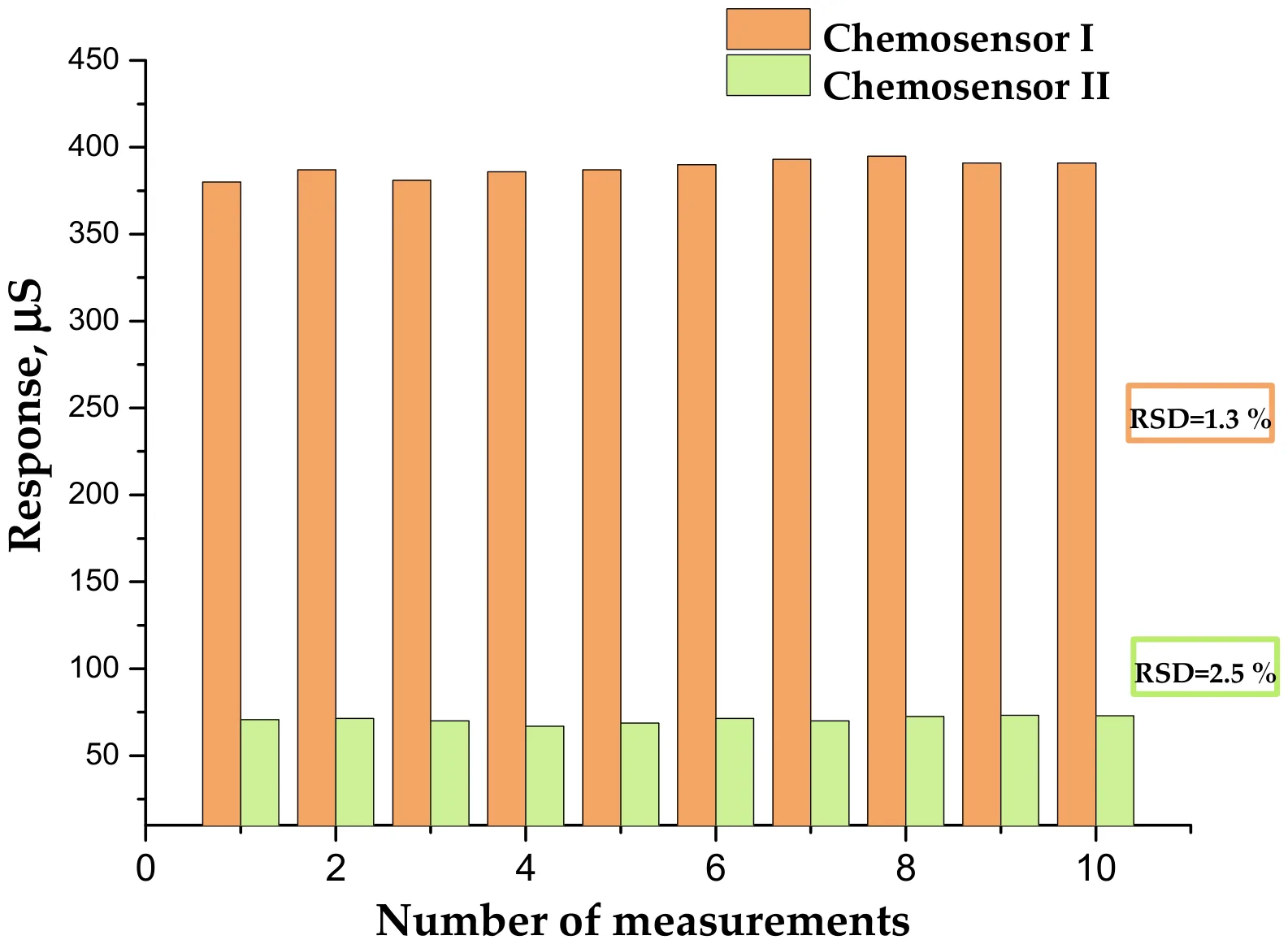
Signal reproducibility of the two chemosensor types measured in 5 mM phosphate buffer (pH 7.5) at an L-arginine concentration of 2 mM.

Signal reproducibility and operational stability are also among the key performance parameters of any sensor. Therefore, in the next stage of the study, the reproducibility of the responses generated by Chemosensors I and II was investigated. To evaluate signal reproducibility toward L-arginine, the sensor responses were measured repeatedly over a certain period of time at intervals of 10–15 min (Figure 9). During the entire experiment, both types of chemosensors were maintained in the working buffer solution.

As shown in Figure 9, both chemosensors demonstrated a high level of signal reproducibility, with measurement errors remaining within acceptable limits (RSD = 1.3% and 2.5% for Chemosensors I and II, respectively).

Table 3 summarizes the analytical performance characteristics of both types of chemosensors, including sensitivity, limit of detection (LOD), linear working range for L-arginine determination, signal reproducibility, and other relevant analytical parameters.

The results indicate that Chemosensor II exhibits a response time nearly twice as long as that of Chemosensor I, its sensitivity toward 1 mM L-arginine is approximately fourfold lower, and its linear detection range is sixfold narrower, with an upper limit of only 0.15 mM. In addition, Chemosensor I demonstrates better signal reproducibility. Therefore, in the context of real-sample analysis, the superior analytical performance of Chemosensor I makes it a more suitable platform for practical applications.

### 3.5. Investigation of the Selectivity of the Arginine-Sensitive Chemosensors Toward Other Types of Amino Acids

In the context of chemosensors, selectivity refers to their ability to discriminate the target analyte from potentially interfering substances present in the sample. The emphasis on selectivity arises from the fact that many analytical applications involve complex sample matrices containing numerous chemical species. Under such conditions, chemosensors must not only detect trace amounts of the target analyte but also provide accurate, reliable, and highly specific analytical responses.

Next, the selectivity of both chemosensors was evaluated toward a series of amino acids. Amino acids exhibiting the highest stability constants in complexation with the calixarene receptors were selected for the study. The sensor responses to 2 mM L-arginine and the selected amino acids were compared. The selectivity factor (S) was calculated as the ratio of the sensor response to L-arginine (v_1_) to that obtained for the corresponding amino acid (v_2_), according to the equation S = v_1_/v_2_. The obtained results are summarized in Table 4.

As shown in Table 4, the two chemosensors exhibited markedly different response patterns. Chemosensor I produced relatively high responses to L-lysine, aspartic acid, and glutamic acid, corresponding to selectivity factors of 1.15, 1.9, and 2.05, respectively. Thus, although the highest response was obtained for L-arginine, discrimination between L-arginine and L-lysine was limited.

Chemosensor II produced a particularly high response to L-histidine, reaching 89.3% of the response to L-arginine and giving a selectivity factor of only 1.1. It also showed a substantial response to L-lysine. Consequently, Chemosensor II exhibited limited discrimination between L-arginine and other positively charged amino acids, particularly L-histidine.

Taken together, the RP-HPLC, DFT calculations and conductometric results indicate that the analytical response is determined not only by the intrinsic affinity of the receptor toward amino acids in solution, but also by the number, accessibility and spatial organization of the phosphonic acid binding sites at the electrode interface. The Chemosensor I receptor, bearing two methylenebisphosphonic acid fragments at the upper rim, provides an optimal binding environment for amino acids containing additional cationic groups. This is reflected in the highest responses toward L-arginine and lysine, which can interact with the receptor through the α-ammonium group and the side-chain guanidinium or ε-ammonium fragments, respectively, forming charge-assisted hydrogen bonds and ion-pair interactions.

Aspartic and glutamic acids give intermediate responses, while amino acids containing nonpolar aliphatic side chains such as leucine and isoleucine show weak responses due to the absence of additional donor, acceptor or charged groups in their side chains. Tryptophan also gives a relatively low response for Chemosensor I, since its bulky indole fragment is mainly hydrophobic and provides only limited hydrogen-bonding capability.

Although the Chemosensor II receptor contains four bisphosphonic acid fragments, the increased number of acidic groups does not lead to improved analytical sensor performance. This may be attributed to a less favorable organization of the receptor layer, partial shielding of the binding sites, stronger intra- or intermolecular hydrogen bonding between phosphonic acid fragments, and less efficient conversion of complex formation into a conductometric signal. As a result, Chemosensor I demonstrates a more favorable combination of sensitivity, linear range and reproducibility for L-arginine detection.

Thus, RP-HPLC, conductometric and DFT data should be considered complementary rather than directly proportional. RP-HPLC confirms the ability of the receptors to bind amino acids in homogeneous solution, whereas conductometric measurements reveal how these recognition events are translated into an analytical signal at the modified gold electrode surface. The preferential response of Chemosensor I toward L-arginine may therefore be attributed to an optimal balance of receptor preorganization, binding-site accessibility, and cooperative multipoint interactions involving the α-ammonium and guanidinium groups of L-arginine under the experimental conditions at pH 7.5.

Overall, Chemosensor I exhibits higher sensitivity, a broader linear detection range, and better reproducibility than Chemosensor II. These characteristics make it the more promising platform for the quantitative determination of L-arginine. However, its application to pharmaceutical, sports-nutrition, cosmetic, and animal-feed samples may require evaluation of the matrix effects and potential interference from L-lysine and other cationic compounds.

### 3.6. Comparison of the Main Analytical Characteristics of the Developed Sensor with Previously Reported L-Arginine Sensors

A comparison of the developed L-arginine sensor with previously reported L-arginine sensors is presented in Table 5.

As summarized in Table 5, a wide variety of electrochemical and optical sensors have been developed for L-arginine determination. Several reported systems exhibit very low limits of detection, including poly-CoTPzPyPc (0.6–2.5 μM) and L/D-CDs-PP (2.39–23.87 μM), but their linear detection ranges are relatively narrow (e.g., 2–60, 10–100, or 0–180 μM), limiting their applicability to samples with a broad concentration range. Other platforms, such as Fe_3_O_4_-SiO_2_-Ag nanoparticles and TPEAM-Cu(II), also provide satisfactory sensitivity but operate within very restricted working ranges (30–60 and 0–80 μM, respectively), while several electrochemical systems show higher detection limits (10–20 μM). Therefore, despite the excellent sensitivity of some previously reported sensors, none combines a broad linear range, good reproducibility, and practical analytical performance as effectively as the proposed system.

The Chemosensor I developed in this work represents one of the most balanced and practically advantageous platforms for L-arginine determination. Although its LOD (5.6 μM) is not the lowest among the reported sensors, it offers one of the broadest linear ranges (5.6–1000 μM), covering both low and high analyte concentrations without sacrificing analytical performance. Moreover, the sensor demonstrates excellent reproducibility (RSD of 1.3%), surpassing or matching most previously reported systems. These analytical characteristics are attributed to the rational molecular design of the calixarene receptor, wherein two methylenebisphosphonic acid groups create well-defined cooperative binding sites for the guanidinium and ammonium groups of L-arginine while minimizing steric hindrance and intramolecular interactions. As a result, Chemosensor I offers a favorable combination of sensitivity, wide working range, and stability, making it a promising platform for practical analysis of pharmaceutical formulations and sports-nutrition samples.

## 4. Conclusions

A new calixarene derivative bearing two 3-(methylthio)propoxy groups at the lower rim and two methylenebisphosphonic acid fragments at the upper rim of the calixarene was synthesized. The phosphonic acid groups act as binding sites capable of interacting with amino acid guests through hydrogen bonding and charge-assisted interactions, whereas the sulfur-containing groups were introduced to promote immobilization of the calixarene receptor on gold conductometric transducers through sulfur–gold interactions. The synthesized calixarene was used as a receptor component of a conductometric sensor system for the determination of L-arginine in aqueous solutions. The key analytical characteristics of the developed chemosensor based on newly synthesized calixarene were investigated. The developed sensor demonstrated a low limit of L-arginine detection (0.0056 mM); a wide linear range (up to 1000 μM); and a high response reproducibility (RSD = 1.3%). The stability constants of the supramolecular complexes formed between Chemosensor I receptor and selected amino acids were determined by high-performance liquid chromatography (HPLC). The method was based on the dependence of amino acid retention, expressed as capacity factors, on the concentration of calixarene in the mobile phase. The calculated stability constants depended on the nature of the amino acid and ranged from logK_A_ = 4.26 for leucine to logK_A_ = 4.52 for L-arginine. Furthermore, density functional theory (DFT) calculations were used to elucidate the molecular interactions underlying the recognition of L-arginine by the calixarene receptor.

The analytical performance of the newly developed sensor was compared with that of a previously developed calixarene-based sensor containing four methylenebisphosphonic acid fragments at the upper rim and two methylthiopropoxy groups at the lower rim of the macrocycle. This comparison allowed evaluation of the effect of the number and spatial arrangement of phosphonic acid binding sites on the recognition of L-arginine. The results demonstrate that the developed chemosensor exhibits improved analytical performance compared with the previously reported chemosensor, including higher sensitivity, a wider dynamic range, faster response time, and improved reproducibility.

Overall, the developed calixarene-based chemosensor provides a simple, rapid, and enzyme-free platform for the conductometric determination of L-arginine over a broad concentration range in aqueous solutions. These characteristics highlight its potential for further application in the analysis of L-arginine in biological, pharmaceutical, and food-related samples.

## Figures and Tables

**Table 1:** Selected hydrogen-bonding parameters for the Chemosensors I and II receptor complexes with L-arginine.

	Donor, D-H	Acceptor, A	H⋯A, Å	D⋯A, Å	∠D–H⋯A, °	ΔE_int_ *
Chemosensor I receptor	O118-H119	O61	1.44	2.47	175.1	−244.8 kJ mol^−1^ (−58.5 kcal mol^−1^)
	N123-H125	O61	1.64	2.65	157.2	
	N115-H117	O59	1.77	2.78	166.8	
	N120-H122	O53	1.88	2.84	153.0	
	O62-H63	O118	1.99	2.90	155.4	
	N111-H114	O53	2.03	2.95	147.4	
Chemosensor II receptor	N108–H111	O64	2.00	3.01	166.8	−269.2 kJ mol^−1^ (−64.4 kcal mol^−1^)
	N112–H113	O57	1.94	2.89	154.0	
	O115–H116	O66	1.63	2.61	163.1	
	N120–H121	O59	1.97	2.93	154.3	
	N120–H122	O140	1.67	2.70	168.5	
	N120–H123	O51	1.72	2.72	158.8	

*—The interaction energy was determined using equation: ΔE_int_ = E_complex_ − E_calixarene_ − E_Arg_.

**Table 2:** Stability constants logK_A_, Gibbs free energies ΔG (kJ/mol) of the Chemosensor I receptor complexes with amino acids, and L-arginine selectivity (S = K_A(Arg)_/K_A(AA)_).

Amino Acid	logK_A_	ΔG	S
Leu	4.260 ± 0.072	−24.26	1.83
Glu	4.282 ± 0.060	−24.38	1.74
Asp	4.284 ± 0.068	−24.40	1.73
Trp	4.306 ± 0.077	−24.53	1.65
His	4.309 ± 0.060	−24.54	1.63
Ile	4.390 ± 0.053	−25.06	1.36
Lys	4.392 ± 0.088	−25.07	1.35
Arg	4.523 ± 0.072	−25.76	-

**Table 3:** Comparison of analytical characteristics of Chemosensor I and Chemosensor II.

Analytical Characteristics	Chemosensor I	Chemosensor II
Sensitivity to 1 mM L-arginine, μS	213.0	48.75
Limit of detection, mM	0.0056	0.005
Linear range, μM	5.6–1000	5–150
Response time, s	80	150
Signal reproducibility (RSD, %)	1.3	2.5
Baseline noise (μS)	0.5	0.375
Baseline drift (μS/min)	0.43	0.0625

**Table 4:** Relative responses and selectivity factors of both chemosensors.

Class of Amino Acid	Amino Acids	* Chemosensor I, Response, %	S	* Chemosensor II, Response, %	S
nonpolar	Ile	8.5	11.8	4.8	20.6
	Leu	8.6	11.6	5.7	17.5
polar negatively charged	Asp	51.3	1.9	5.8	17.2
	Glu	48.6	2.05	1.6	62.5
polar positively charged	His	13.6	7.4	89.3	1.1
	Lys	85.0	1.15	50.7	2.0
	Arg	100	-	100	-
aromatic	Trp	10.4	9.6	15	6.7

* The response to 2 mM L-arginine was normalized to 100%.

**Table 5:** Comparison of the main analytical characteristics of L-arginine sensors.

Bioselective Element	Measurement System	LOD, µM	Linear Range, µM	Reproducibility (RSD, %)	Ref.
poly-CoTPzPyPc	Amperometric	2.5 (CV) 0.6 (CA)	10–100 (CV) 2–60 (CA)	1.63	[40]
TPEAM-Cu (II)	Fluorogenic	4	0–80	2.5	[41]
MCPE	Amperometric	4.3	20–263	4.2	[42]
L/D-CDs-PP	Ratiometric Fluorescence	23.87 (L) 2.39 (D)	0–600 (L) 0–180 (D)	<4.39% (L) <1.37% (D)	[43]
Fe_3_O_4_-SiO_2_-Ag NP	Colorimetric	2.506	30–60	n/a	[44]
rGO + ARG + URE	Potentiometric	10	10–1000	n/a	[45]
BSA-CDs	Fluorescent	25.8	400–4500	n/a	[46]
ADI/Fe_3_O_4_/MWCNT/PANI/GCE	Amperometric	20	100–1000	n/a	[47]
calix[4]arene with 4 bisphosphonic acid groups	Conductometric	5.0	5–150	2.5	[31]
calix[4]arene with 2 bisphosphonic acid groups	Conductometric	5.6	5.6–1000	1.3	This work

Note: poly-CoTPzPyPc—pyrazolopyrimidinium analog bearing cobalt (II) phthalocyanine polymer, CA—chronoamperometry, CV—cyclic voltammetry, TPEAM—triphenyl ether amide, MCPE—copper microparticle-modified carbon paste electrode, L/D-CDs-PP—levorotatory/dextrorotatory carbon dots and porphyrin, NPs—nanoparticles, rGO—reduced graphene oxide, ARG—arginase, URE—urease, BSA-CDs—bovine serum albumin-derived carbon dots, ADI—arginine deiminase, MWCNTs—multiwalled carbon nanotubes, PANI—polyaniline.

## Data Availability

The raw data supporting the conclusions of this article will be made available by the authors on request. The authors have reviewed and edited the output and take full responsibility for the content of this publication.

## Supplementary Materials

The following supporting information can be downloaded at: https://www.mdpi.com/article/10.3390/mi17091047/s1, Figure S1. ^1^H and ^31^P NMR spectra of calixarene bisphosphonates **2** (CD_3_OD); Figure S2. ^13^C NMR spectrum calixarene bisphosphonates **2** (CD_3_OD); Figure S3. ^1^H NMR spectra of calixarene bisphosphonates **3.1** (a) and **3.2** (b) (CDCl_3_); Figure S4. Two-dimensional HMBC NMR spectrum of calixarene methylenbisphosphonate **3.1**; Figure S5. ^13^C NMR spectrum of calixarene bisphosphonates **3.2** (CDCl_3_); Figure S6. ^1^H and ^31^P NMR spectra of 5,17-Di(bis(dihydroxyphosphoryl)methyl)-25,27-bis(3-(methylthio)propoxy)calix[4]arene **4**; Figure S7. ^13^C NMR spectrum of calixarene methylenebisphosphonic acid **4** (CD_3_OD); Figure S8. Retention time of L-Arginine before (a) and after (b) addition of Chemosensor I receptor to the mobile phase (concentration of Chemosensor I in the mobile phase: 0.8 × 10^−4^ M) [48,49,50]; Figure S9. The dependences of (1/k’) values of the amino acids on the concentration of Chemosensor I receptor in the mobile phase [51]; Figure S10. Responses of Chemosensor I to L-arginine, L-isoleucine, L-histidine and L-lysine.

## Abbreviations

The following abbreviations are used in this manuscript:

L-Arg	L-Arginine
DMSO	Dimethyl Sulfoxide
THF	Tetrahydrofuran
DFT	Density Functional Theory
PPh3	Triphenylphosphine
DEAD	Diethyl Azodicarboxylate
RP HPLC	Reversed-Phase High-Performance Liquid Chromatography
CPCM	Conductor-Like Polarizable Continuum Model
SMD	Solvation Model Based on Density
NMR	Nuclear Magnetic Resonance
HMBC	Heteronuclear Multiple-Bond Correlation
