# Supplementary material for: Novel Conductometric Chemosensor Based on Methylenebis(Phosphonic Acid)-Modified Calixarene for L-Arginine Determination
Source: Micromachines (Basel). 2026 Sep 2;17(9):1047. doi: 10.3390/mi17091047 (PMC13609174; doi:10.3390/mi17091047)
Supplement: Supplementary file 1 [file micromachines-17-01047-s001.zip › micromachines-4469179-supplementary.pdf]

Supplementary Materials

Novel Conductometric Chemosensor Based on Methylenebis(Phosphonic Acid)-Modified Calixarene for L-Arginine Determination

Svitlana V. Marchenko [1], Sergiy O. Cherenok [2], Kseniia O. Berketa [1], Veronika A. Bakhmat [1], Olha V. Soldatkina [1], Anna I. Selikhova [2], Olga I. Kalchenko [2], Galyna P. Volynets [1], Oleksandr O. Soldatkin [1,3], Vitaly I. Kalchenko [2], Sergei V. Dzyadevych [1], Abdelhamid Errachid [4,*] and Viktoriya M. Pyeshkova [1,5,*]

[1] Institute of Molecular Biology and Genetics, The National Academy of Sciences of Ukraine, 150 Zabolotnoho Str., 03143 Kyiv, Ukraine
[2] Institute of Organic Chemistry, The National Academy of Sciences of Ukraine, 5 Academician Kukhar Str., 02094 Kyiv, Ukraine
[3] National Technical University of Ukraine "Igor Sikorsky Kyiv Polytechnic Institute", Department of Translational Medical Bioengineering, 37 Beresteysky Avenue, 03056 Kyiv, Ukraine
[4] Unité Mixte de Recherche 5280, Institut des Sciences Analytiques, Université Claude Bernard Lyon 1, Centre National de la Recherche Scientifique, 5 rue de la Doua, 69100 Villeurbanne, France
[5] School of Engineering, University of Warwick, Coventry CV4 7AL, UK
* Correspondence: abdelhamid.errachid-el-salhi@univ-lyon1.fr (A.E.); victoriya.p@gmail.com (V.M.P.)

NMR spectra the calixarenes

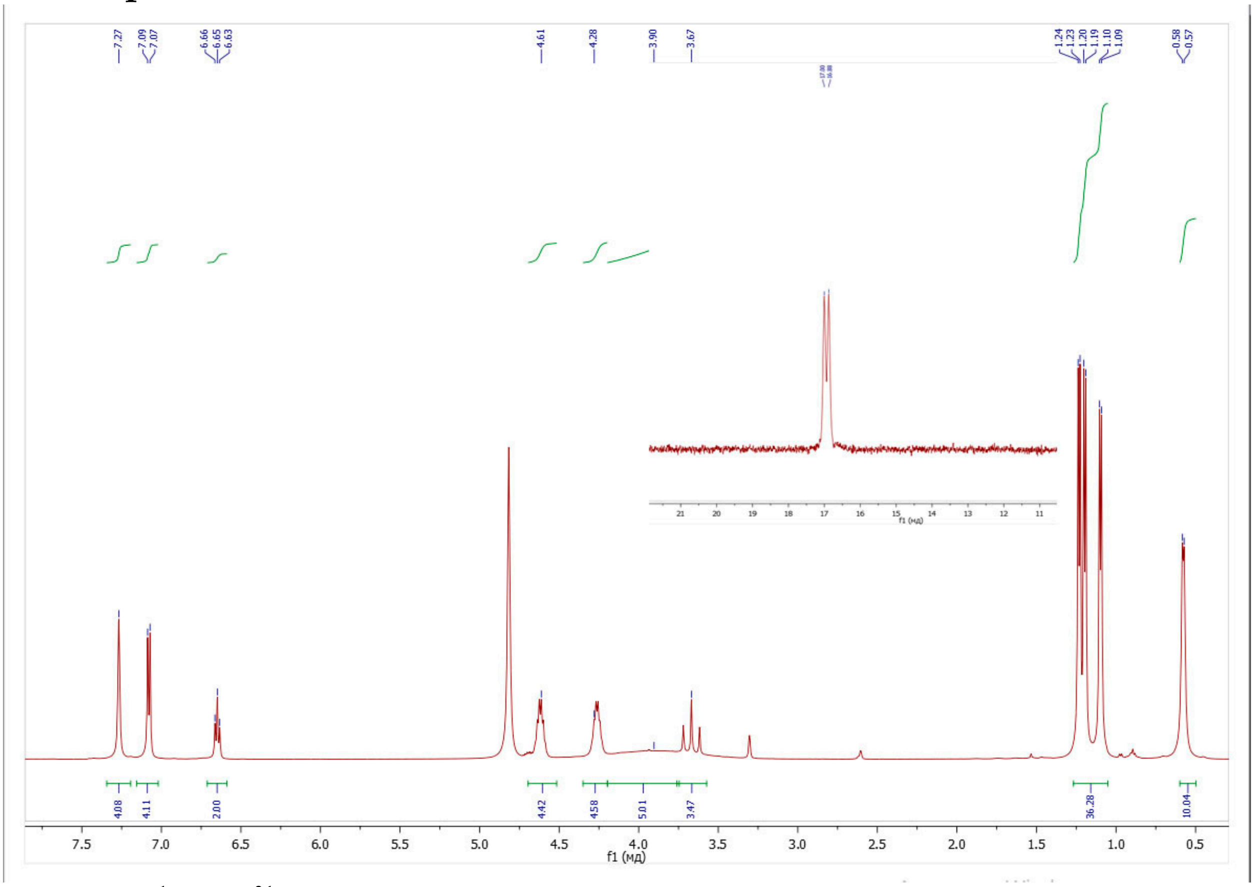

Figure S1. ^{1}H and ^{31}P NMR spectra calixarene bisphosphonates **2** (CD_3OD);

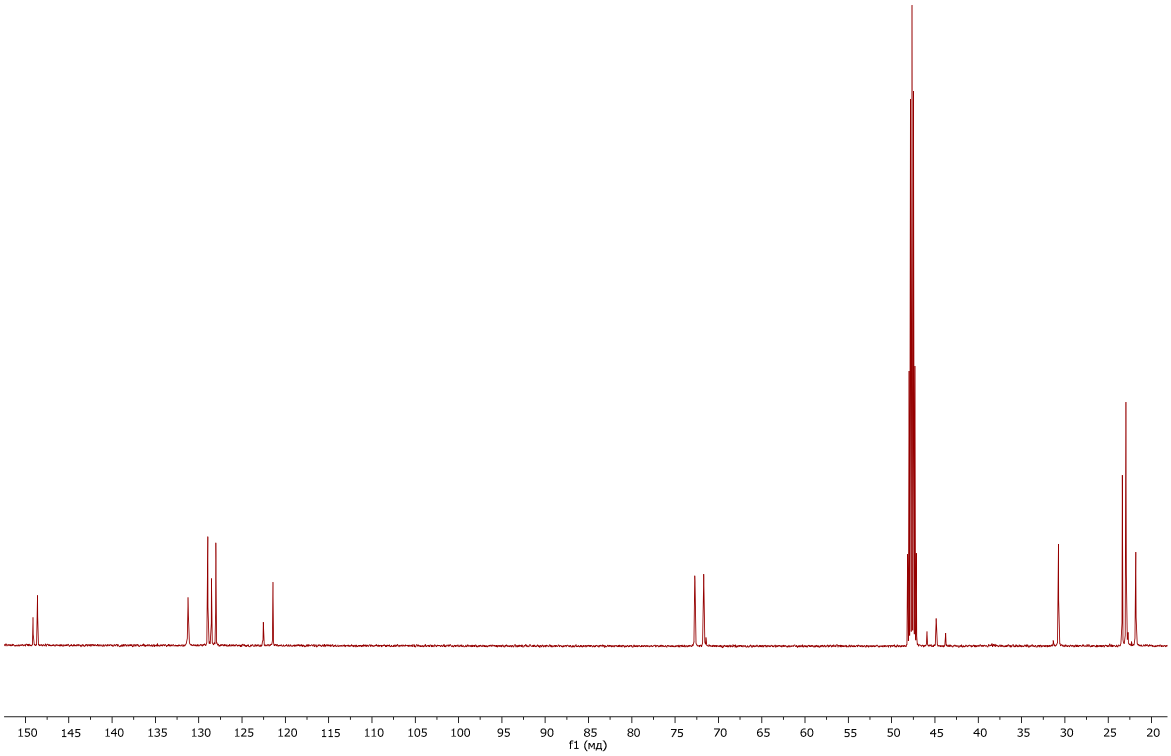

Figure S2. ^{13}C NMR spectra calixarene bisphosphonates **2** (CD_3OD)

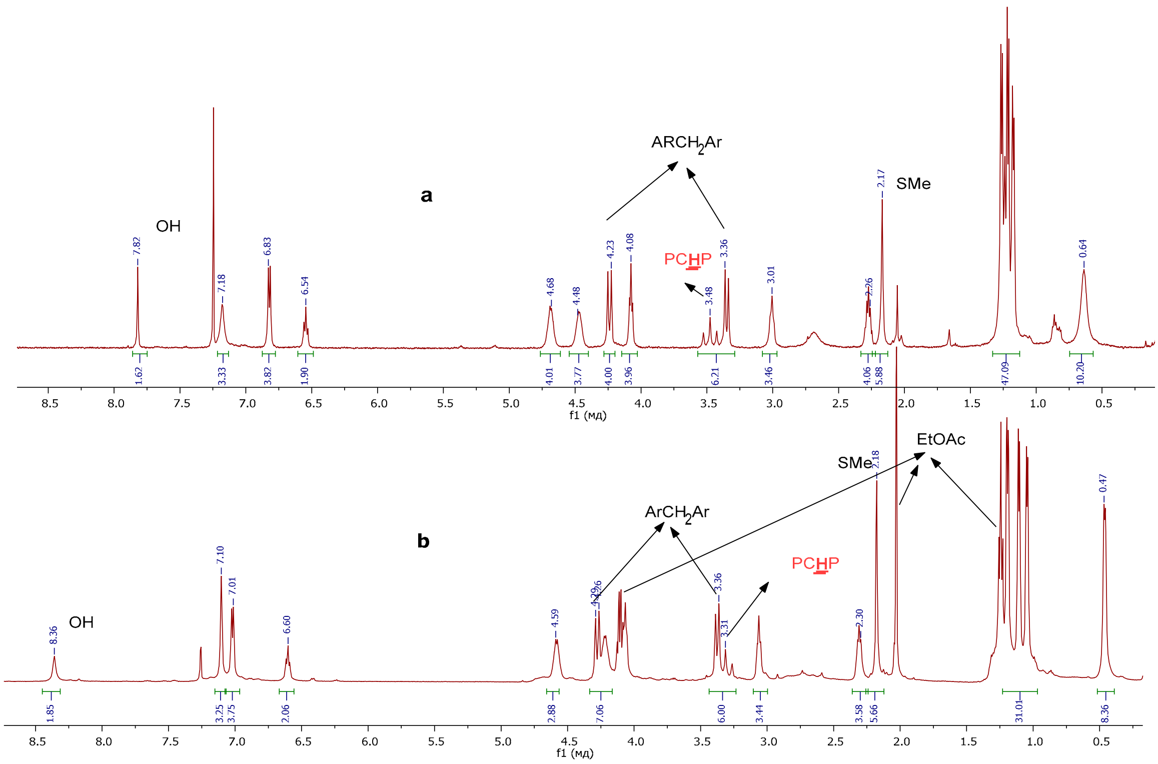

Figure S3. ^{1}H NMR spectra calixarene bisphosphonates **3.1** (a) and **3.2** (b) ($CDCl_3$)

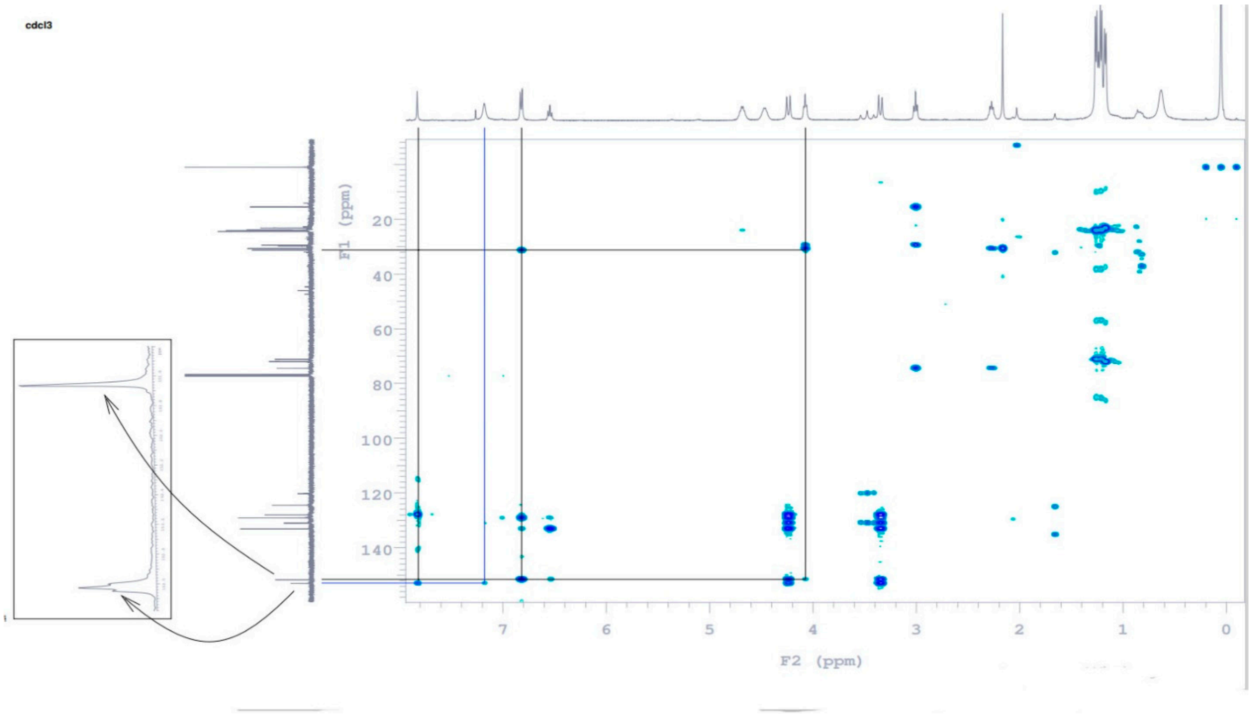

Figure S4. Two-dimensional HMBC NMR spectrum of calixarene methylenbisphosphonate **3.1**

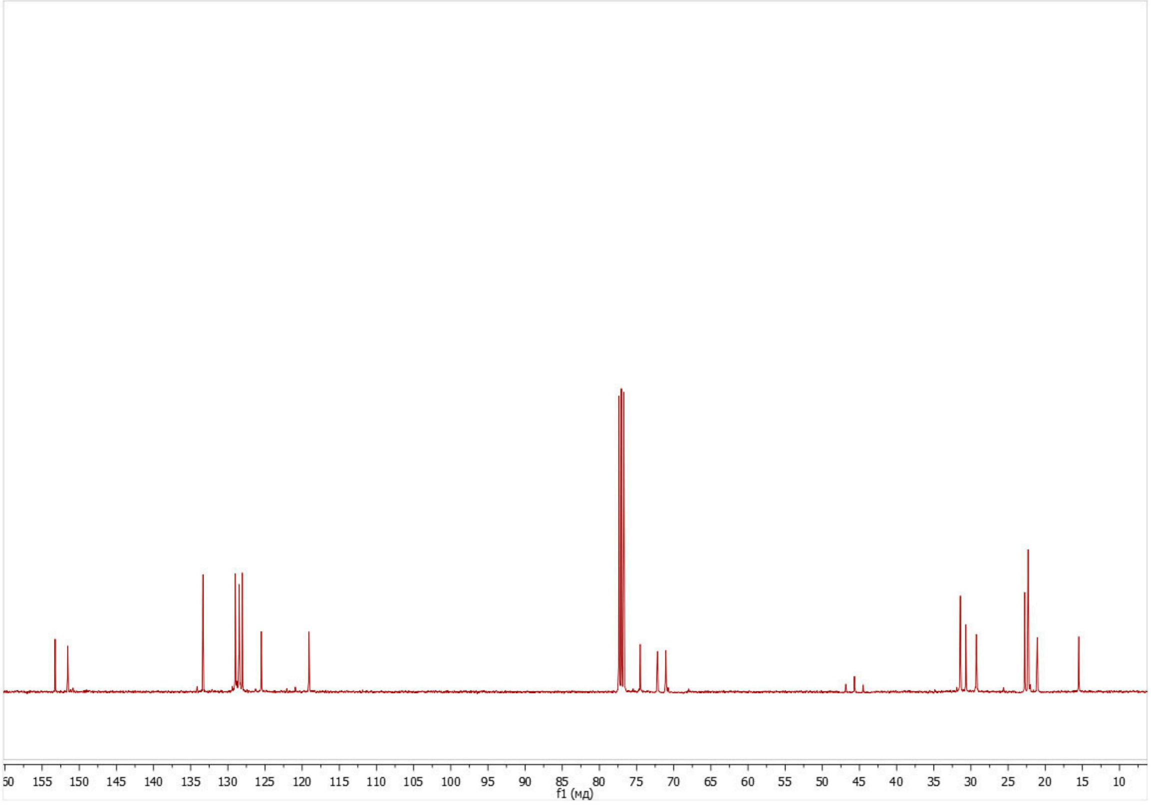

Figure S5. ^{13}C NMR spectra calixarene bisphosphonates **3.2** ($CDCl_3$)

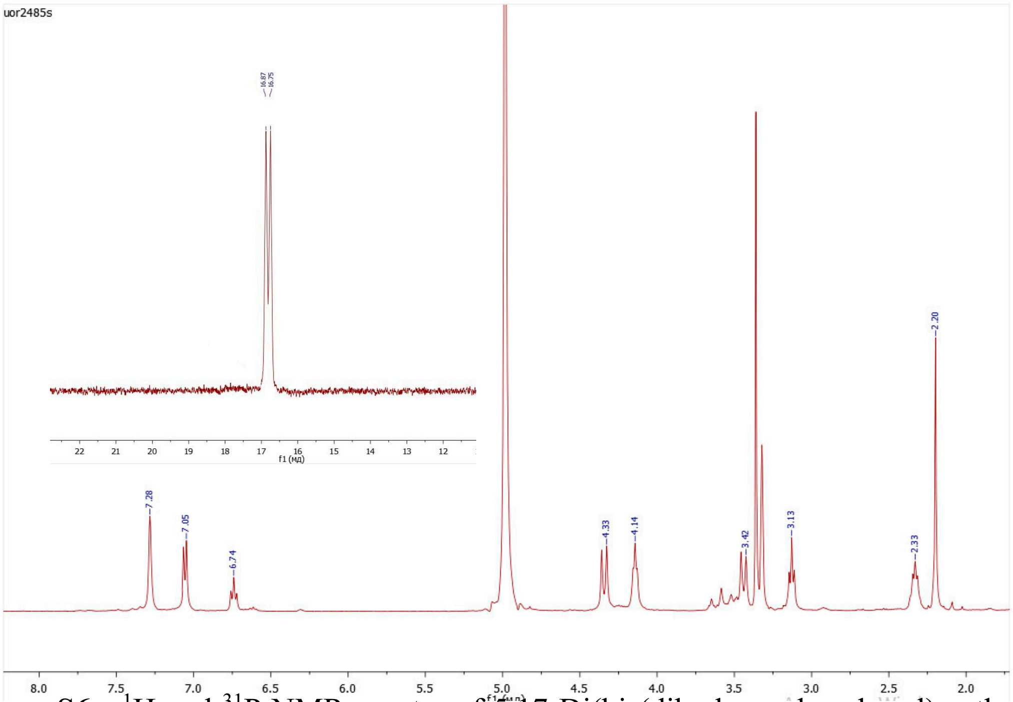

Figure S6. ^{1}H and ^{31}P NMR spectra of 5,17-Di(bis(dihydroxyphosphoryl)methyl)-25,27-bis(3-(methylthio)propoxy)calix[4]arene **4**

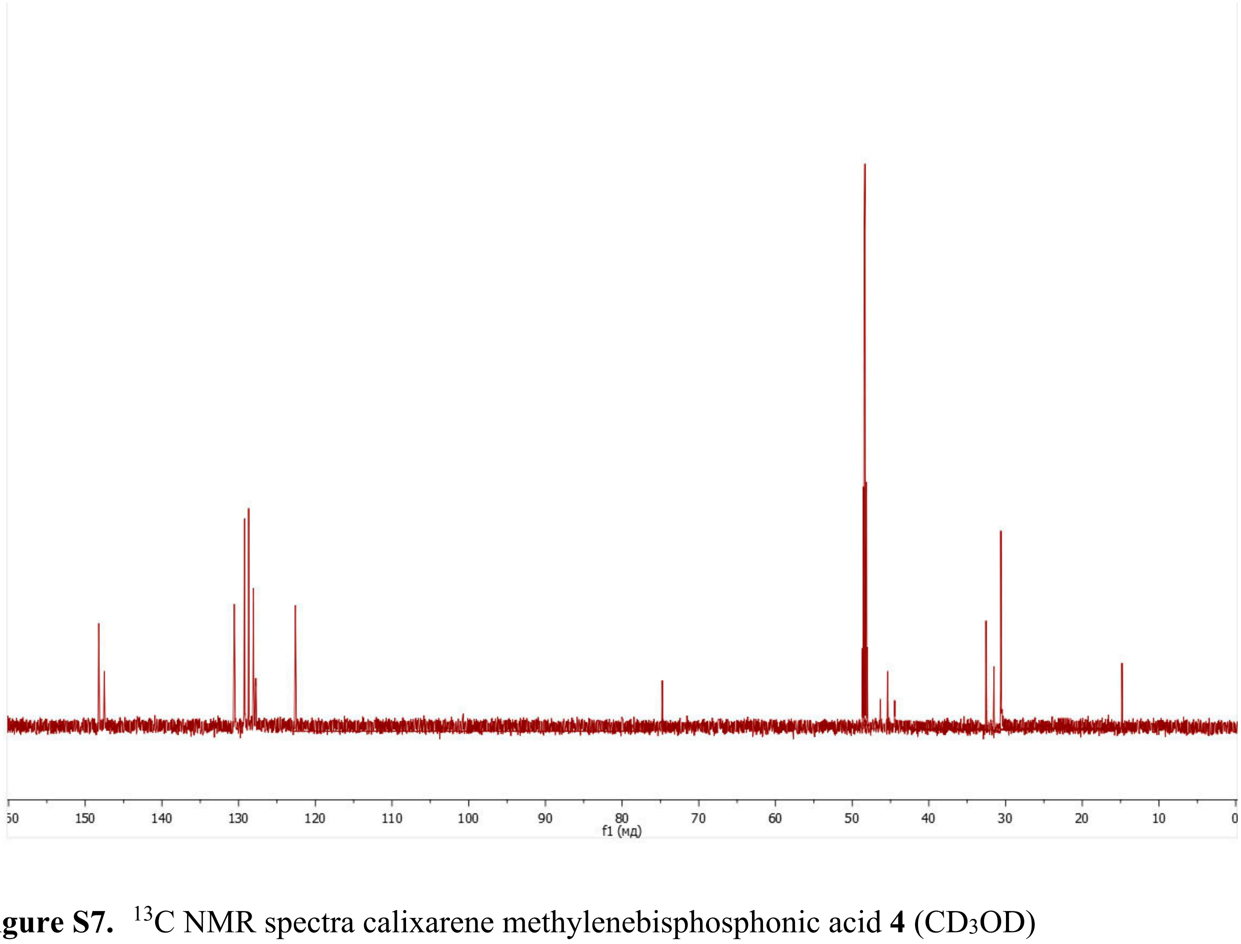

Figure S7. ^{13}C NMR spectra calixarene methylenebisphosphonic acid **4** (CD_3OD)

DFT analysis of the L-arginine complexes

Cartesian coordinates of the Chemosensor II receptor–L-arginine complex

Coordinates from ORCA-job c192Arg2 E -6919.049752345113

C	-2.78794960120530	-1.99966986062832	1.60096829117767
C	-2.17497650307975	-3.46464367994404	-0.69582895581388
C	-1.76135086481135	-2.94486758873281	1.60787661709185
C	-3.48115728247627	-1.75834160623654	0.40080275651953
C	-3.18894922198896	-2.51080696530359	-0.75302922488309
C	-1.43885455468451	-3.68495508388391	0.46715975496756
H	-1.18033027785857	-3.07355880829525	2.52379316299106
H	-1.92450804680979	-4.02397438090863	-1.60206272724696
C	-3.09266278510215	-1.20160416594310	2.85785769304727
H	-3.03128608678471	-1.87948352434838	3.72092338459350
H	-4.12238713636314	-0.83246580657623	2.81115518953351
C	-2.11232178964183	-0.06277801457934	3.07679023953229
C	-0.13823590778696	1.92587437212908	3.24525332001381
C	-2.37130730739596	1.24832472628720	2.64439480700089
C	-0.87376502936745	-0.32253771930561	3.66686168784149
C	0.11381193402561	0.65971788041288	3.77852511847781
C	-1.38166411479327	2.24099901298025	2.68850902434915
H	-0.66791780797604	-1.32865431470160	4.04355494625578
H	0.63323924489730	2.69941801025435	3.27427512297231
O	-4.43754380286213	-0.79593523927861	0.39602398495223
O	-3.59693410674489	1.56676739703974	2.10573504524722
C	-3.84277872821204	-2.21483735601711	-2.09028515983061
H	-3.92179369410260	-3.14730574112341	-2.66455917883000
H	-4.85998990394510	-1.82487006922386	-1.95306864645668
C	-2.96901152440809	-1.23436351096010	-2.84592199186575
C	-1.07407303762165	0.54841340486795	-3.87122741307041
C	-1.91722369996985	-1.69513117764333	-3.63546661746501
C	-3.10440012850146	0.14703281153191	-2.65149094406922
C	-2.15004284222414	1.05601471743427	-3.13274492206326
C	-0.98026252592508	-0.81340699281262	-4.17782352902254
H	-1.82073686847041	-2.76792083597263	-3.82466320128974
H	-0.30733194794675	1.24246728902013	-4.22569981561216
C	-1.60549397047112	3.60637118427708	2.06486619895844
H	-2.66329830499760	3.89134314753771	2.12748281563074
H	-1.03433148015986	4.35229986331076	2.63310112140434
C	-2.26356074209295	2.55086095838106	-2.87980760092420
H	-1.94787581941038	3.06960401933287	-3.79596521145035
H	-3.31337754606662	2.81571319648248	-2.70552641211281
C	-1.41585548660441	3.06817391847727	-1.73074754141485
C	0.16728433483293	4.03955836116200	0.34468398547865
C	-0.09342079780428	3.46157561475395	-1.96121142145279
C	-1.93426174949401	3.14921694877512	-0.42839286006294
C	-1.12932188767787	3.62355968855068	0.62639470781361
C	0.71873310302104	3.95340217255115	-0.93751218369854
H	0.28324035373464	3.36188115828313	-2.98106477880234
H	0.77274708461568	4.42362303800265	1.17025733788663
O	-3.22568814939354	2.79397103486276	-0.23934655769196
C	2.16974315603228	4.36852343635826	-1.12778980973542

H	2.28093717419334	5.44864623133171	-0.92599364572254
P	3.28182926671076	3.54374217109666	0.09890854261588
O	2.61603138608707	2.27096499010841	0.61994450297089
P	2.83707346785520	4.17111346145783	-2.81385591872407
O	2.71990800485487	2.78707724014790	-3.36404875998747
C	-0.19286093104121	-4.54248881678322	0.38528364757720
H	-0.18564544106307	-5.08318190815103	-0.57620971064971
P	0.01466416658925	-5.86231030866338	1.61226364794077
O	1.40623258203680	-6.40211194092243	1.68639287369000
P	1.25353201375241	-3.42411227535129	0.32759795852652
O	0.98642085233773	-2.29832515370395	-0.67187245488654
O	-1.11683457130510	-6.89193632955206	1.12762278327728
H	-1.18787568902502	-7.67954286869038	1.69210301800158
O	-0.43332041489197	-5.36297727471217	3.07215269645811
H	-1.39178627222546	-5.26665459538677	3.19036682209457
O	2.48234565531349	-4.35291143308885	-0.20205491424586
H	2.54213707343390	-4.38789554695002	-1.17011052684842
O	4.67063651986181	3.38075534022697	-0.52616091391092
O	3.34984708567021	4.52620580851451	1.37941538573057
H	3.86587373077493	5.33235383071320	1.22056620953145
O	2.11011769206538	5.29606642567342	-3.70108320314733
H	1.50872825126665	4.91534492976355	-4.36014985691642
O	4.31025919823676	4.78376235987190	-2.72495443892773
H	4.75768433580980	4.35786644585088	-1.94692531769864
H	-3.40166148921485	2.44660602972482	0.66286808826214
H	-4.44590302996123	-0.31767891738529	-0.46163836006376
C	-4.71222118062105	1.67472701802621	3.01103419429777
H	-5.57018194519510	1.21841284591361	2.49279463595625
H	-4.49389748823388	1.07394872014668	3.90654358435737
C	-5.01098454484490	3.11056931321614	3.39886173622328
H	-5.77057784621266	3.07212432669582	4.19697919172300
H	-4.10886666786340	3.54953717033732	3.85821125297427
C	-5.51209717314369	3.97578517137778	2.24959715813749
H	-6.47017605253442	3.59745026911610	1.85988001960346
H	-4.80071102894024	3.99489933096068	1.41000741055654
H	-5.67023182145747	5.01512849895424	2.57397257008920
O	-4.18660518648610	0.60183837497535	-1.93401383516035
C	-5.35578207315464	0.92653533223031	-2.70610000295901
H	-5.03238417016278	1.31856864209768	-3.68346954480432
H	-5.92875830644392	-0.00078425860948	-2.88582729999758
C	-6.18878090770735	1.95268941991458	-1.96704198169328
H	-5.61942248971969	2.89449662328470	-1.90036855806404
H	-7.06516657267882	2.16187911761060	-2.60183738880102
C	-6.63848971030442	1.51661314161651	-0.57834962576018
H	-5.78662904710852	1.44131445086417	0.11359545322629
H	-7.13807860906840	0.53489919861630	-0.60590251763120
H	-7.34700319598690	2.24138648243993	-0.15074837340425
C	4.12024065288234	-0.25933498223533	-0.77655892282559
C	4.92841441554831	0.15603157769422	-2.01524381551712
O	5.23565007116998	-0.65027780665657	-2.86753359989148
H	4.46466119609119	0.32627851159104	0.08961015679777
C	4.24173280557234	-1.74799082209006	-0.50967738587730

H	3.85803022374906	-2.28130640645620	-1.39062160998211
H	3.55083646483796	-1.95537051309594	0.31605735234856
C	5.64121115495025	-2.24073626863236	-0.11959211425303
H	6.39785133565363	-1.45587376447191	-0.27338687984542
H	5.94028006721740	-3.08053600145607	-0.76465738757237
C	5.72265848768346	-2.67073616464194	1.33799051681179
H	6.75552136914347	-2.96707456460500	1.58034787259714
N	4.81977203183468	-3.78739290685028	1.60733845294770
H	5.45627134199133	-1.81924055485717	1.98802862770948
C	4.60855617121949	-4.27962625979756	2.82832449944983
H	4.13061572637449	-4.04190768585285	0.89605761333742
N	3.68050151888293	-5.21388665874321	3.02951790274834
H	2.98914904959052	-5.47481493000713	2.32072943801284
H	3.50526808697858	-5.52762625710309	3.97693258212686
O	5.28515511018318	1.41504914402607	-2.12917212965110
H	4.99782367019335	2.03618715156916	-1.39896932503768
N	5.38068550216587	-3.89510102583580	3.84818881205524
H	5.98258107484841	-3.08602961907829	3.75645750458466
H	5.15080353767146	-4.20224164678435	4.78616207307838
N	2.68499589042974	0.07880218223726	-0.98801310003047
H	2.07992421987412	-0.66089532186054	-0.58857639731227
H	2.43611663170592	0.11511706522463	-2.00611362746557
H	2.46688002817535	0.97605250613284	-0.50478385167096
C	1.42910933149524	0.28909819570513	4.43578032298254
H	1.30402221622672	-0.66677662162148	4.97087004413716
P	2.77314869906238	-0.06487574527049	3.23476027285611
O	3.79066179726542	-0.99263186501871	3.81846135781899
P	2.00777646932157	1.42493184630579	5.73918697196701
O	3.05064070624244	0.80062266591687	6.59952694684489
O	2.05801515583589	-0.54307748735673	1.90762488221387
O	3.36891402355286	1.36817653110723	2.84782320313736
H	3.06370619252123	1.73495154588902	1.93922979649544
O	2.43347612548037	2.78921617515278	5.00172792541757
H	2.91644412208725	2.58996005125212	4.16837131499462
O	0.73880424861742	1.87137101551763	6.60717238977887
H	0.10698830688200	2.44644433254714	6.14654284962905
C	0.12301924419006	-1.37875727425311	-5.05189223060175
H	-0.11613632668839	-2.42959923665884	-5.29139555392084
P	1.72296193730021	-1.49001994887029	-4.17190903954069
O	2.13297611989713	-0.14918870282134	-3.62733499706145
P	0.29439959088540	-0.64858430953460	-6.70740474415723
O	1.58084554621405	-1.08359890226403	-7.34639952164146
O	2.73730663989422	-2.13399404987573	-5.21929319157213
H	2.52462552347144	-1.81156535608227	-6.13706353431974
O	1.58077848813318	-2.65173815416171	-3.11489631301267
H	1.30467743830384	-2.43241725761598	-2.15728378631270
O	-1.06208214182057	-1.13357303344955	-7.39782732546513
H	-1.20591793073681	-0.78360127732728	-8.29350400717035
O	0.24874181136807	0.95059178922778	-6.66583940242244
H	-0.63356862746781	1.33814272788024	-6.54512958397696
O	1.59953714101888	-3.00240029564093	1.75033244626901
H	1.86204356664689	-1.55561434160615	1.87666540627051

Cartesian coordinates of the Chemosensor I receptor–L-arginine complex

Coordinates from ORCA-job c940arg2 E -4570.891965903100

C	-2.08091967319007	-1.99211556234330	1.57433007537108
C	-1.54802821744578	-2.96321562838610	-0.98076442814495
C	-0.89849308668974	-2.68300836649638	1.30704188580156
C	-2.96197935789864	-1.70997297971911	0.51329692572891
C	-2.70223819592992	-2.20826081342519	-0.77481398526576
C	-0.61430868484100	-3.17820216661046	0.03284110971203
H	-0.19091645399333	-2.83520065018006	2.12618203412360
H	-1.33944031097813	-3.34840320501484	-1.98212810183856
C	-2.36362113164389	-1.52868264807023	2.99367946717532
H	-2.07708503774736	-2.33291776159596	3.68508754221093
H	-3.44123601657476	-1.35847386508337	3.12463733739940
C	-1.57863284339661	-0.28419385498644	3.34453346620331
C	-0.02368241766597	2.01133050693336	3.74376597973735
C	-2.02931554582279	0.97631287997868	2.92991469867692
C	-0.34390831721719	-0.36647275837491	3.99456942289956
C	0.41698766401372	0.77939040611557	4.21966015782239
C	-1.24660508352875	2.13015710342724	3.07303055785648
H	0.01811695577453	-1.34193204400818	4.33276158736259
H	0.59431120239292	2.90214025653402	3.88508320917078
O	-4.08173921714161	-0.96266876842692	0.71258976219455
O	-3.27814154218919	1.07143688265883	2.34780229837066
C	-3.56241265638760	-1.83273999843453	-1.96974397862502
H	-3.74171885816406	-2.72621562555869	-2.58439345969244
H	-4.53543338886637	-1.45732309321669	-1.63310937106337
C	-2.82441174454474	-0.79953222862131	-2.80032848259675
C	-1.14873318557886	1.07534773838036	-4.05510379010529
C	-1.89257255695008	-1.19971326880987	-3.76477572598172
C	-2.93388306837791	0.57083407964196	-2.52617982925194
C	-2.08657502991207	1.51760779776559	-3.11734846036286
C	-1.06665273637593	-0.27268544440481	-4.39486954953387
H	-1.80209373955773	-2.26167136048938	-4.00820273809690
H	-0.47941945021462	1.80217442264176	-4.52399760747271
C	-1.63216435428917	3.43254607223166	2.39922371727129
H	-2.72030535374179	3.55684336542954	2.36238479255881
H	-1.21917048028409	4.27069947926857	2.97813188202761
C	-2.11389877395663	2.96167757837036	-2.66658512998809
H	-1.70840411085700	3.58983959664461	-3.47143841244603
H	-3.14588778623092	3.29357825657921	-2.48685243951769
C	-1.27510857360976	3.17900437805256	-1.41948235417777
C	0.31611048279893	3.65064296608010	0.82152248710610
C	0.10849506668939	3.30779647914102	-1.54220602094652
C	-1.85382071347091	3.22055503277592	-0.13677706880443
C	-1.05612492593467	3.46718676932272	0.99425353486927
C	0.91883227921125	3.55425533313259	-0.43269103494899
H	0.55503490959266	3.21249760969831	-2.53582804277026
H	0.94143079214161	3.83852372090738	1.69909140765860
O	-3.19068587980211	3.04027393697139	0.03441104725536
H	1.37035308496759	0.70864450997064	4.74847863286310
H	-0.33998642173196	-0.60681467396205	-5.13915595737020
C	2.42634395569137	3.63837036701517	-0.53023415494714

H	2.80790547396422	4.30287991165149	0.26116930192203
P	3.18844466940876	1.99747987884483	-0.17186938854007
O	3.38558362029721	1.88250634977268	1.32827784557002
P	3.05108194866512	4.36356433403267	-2.07273438713227
O	2.91001355892304	3.47863485346914	-3.26972670576650
C	0.70732111229702	-3.83185387504899	-0.32557612619557
H	0.54233835499960	-4.57556262630647	-1.12494777815316
P	1.41723591163062	-4.81128471744502	1.02603524349002
O	1.86378527885029	-4.04832200629586	2.23594818107438
P	1.82086804519376	-2.60499399848154	-1.14022627388190
O	3.15301563040096	-3.34621180079450	-1.41109387421349
O	2.54398867478879	-5.75454647609834	0.38433042602160
H	3.45193901888895	-5.40656450297566	0.45557626414045
O	0.26821238052841	-5.84793138825375	1.43933494572656
H	-0.15213370074280	-6.30337257060244	0.69266892471829
O	1.16059189280258	-2.02083245080183	-2.36112398606690
O	2.03169607002501	-1.50816360593912	0.01478937324644
H	2.20499951633205	-0.58007317359248	-0.33882119874581
O	2.46409760488446	0.89082340350239	-0.92009321953787
O	4.69569215555124	2.18120163074043	-0.82965242611471
H	4.74891221427385	1.77101603590641	-1.70769651098662
O	2.36455934562275	5.80769787637429	-2.21201168176239
H	1.58047018155677	5.79072341523923	-2.78242894673886
O	4.54386723313935	4.84103569277974	-1.72722373712299
H	5.04444529421258	4.08674294033868	-1.35916928347911
H	-3.50001976608572	2.32624960616532	-0.56603803313510
H	-3.86293107141875	-0.22177708172095	1.31862928019450
C	-4.31145143042779	1.45422352492979	3.26951947298006
H	-4.41957862601707	0.66481576961885	4.03483427473694
H	-4.00374403496918	2.37986674521154	3.78533847324003
C	-5.60126939082067	1.66348583846268	2.50814631029754
H	-5.42324099929696	2.41567419826137	1.72315785220631
H	-5.86945355457960	0.72476993050455	1.99592995123861
C	-6.73966208388081	2.10244336224161	3.41831335274379
H	-6.51138152387638	3.05682505078260	3.91844076782477
H	-6.93979510818441	1.35401661480011	4.20130803363226
H	-7.66808792400385	2.24039038795437	2.84512242598200
O	-3.88837870489601	1.00899931619698	-1.62837882561823
C	-5.18334942620419	1.21085218949592	-2.21661781274593
H	-5.10273667936151	1.98620732089333	-2.99919899059497
H	-5.50389542308370	0.27420488847065	-2.70415259377157
C	-6.15711637018680	1.61637011460432	-1.13265197213418
H	-6.13721246410579	0.84558081293419	-0.34599799387248
H	-5.80602921595277	2.55381067341119	-0.67080260558925
C	-7.57117201120235	1.79387809883082	-1.66720650124462
H	-7.95792199031087	0.85811859852112	-2.10067735696971
H	-7.61322392047750	2.56785465501612	-2.44971099912915
H	-8.25814537001696	2.09628856147321	-0.86321609429684
C	6.48785905224841	-3.35369090613599	-1.82889249691499
C	6.24627065852324	-4.15322045189621	-0.54657372228580
O	7.19968304408874	-4.57536928860303	0.07022816325761
H	7.37959869840368	-3.79815451912295	-2.28680705979177

C	6.78138896972214	-1.87834929184354	-1.53011041881757
H	6.90825727523022	-1.38101299221896	-2.50341214146399
H	7.76954643348514	-1.84434373747143	-1.04594654679032
C	5.74690293127689	-1.13468309313586	-0.67522482273378
H	4.75453227523168	-1.60808247387874	-0.73570205093163
H	5.61249787349354	-0.11907890190927	-1.06798826747692
C	6.16489142728940	-1.02042255573925	0.79205557602204
H	6.50590362734514	-1.98494007001359	1.19752734187022
N	5.11540809957288	-0.48643537285542	1.64174181334602
H	7.03335214313722	-0.35217250908337	0.87776580898357
C	4.12739503021239	-1.22489332560941	2.16259440608744
H	4.86608851498609	0.49779205742092	1.51331238847865
N	4.21274277509163	-2.54988744089873	2.25098549813253
H	4.91365961311413	-3.03746744475569	1.70458067241395
H	3.34461731363710	-3.08283889367900	2.39417604368711
O	5.01976835820594	-4.30746831874089	-0.09483328286543
H	4.24398721089452	-3.86153668724810	-0.61840796810202
N	3.08122934321408	-0.58568332670837	2.70001559102914
H	2.25445679429348	-1.13741397372624	2.90351599484104
H	2.93193153053525	0.37370200751544	2.36687644844116
N	5.39161287782760	-3.49368234262375	-2.82414081655632
H	5.57512193137286	-2.88779069542269	-3.63193661477753
H	4.43965161232053	-3.25352963994802	-2.43108394160247
H	5.33363671757085	-4.45774287819384	-3.17195488840461

Determination of stability constants K_A of the complexes by HPLC

A convenient and rapid method for determining stability constants (K_A) of supramolecular host-guest complexes of calixarenes with organic molecules in aqueous or aqueous-organic solutions is reversed-phase high-performance liquid chromatography [48,49,50]. According to this method, the stability constants (K_A) are determined from the dependence of the retention time (t) or capacity factor (k') of the guest molecule on the concentration of calixarene in a mobile phase. The addition of calixarene **4** to the mobile phase water/acetonitrile/methanol (80/15/5 by volume) (Zorbax CN column) reduces the retention time (t) of the amino acids (see fig. S8 as an example) due to the formation of the supramolecular complexes.

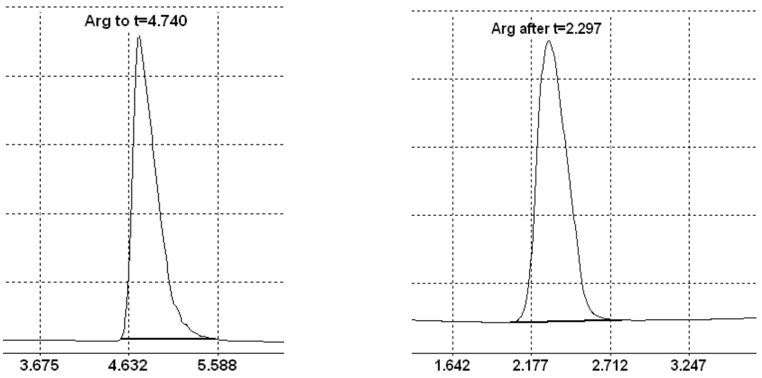

t=4.740 min t=2.297 min

a **b**

Figure S8. Retention time of L-Arginine before (a) and after (b) addition of Chemosensor I receptor to the mobile phase (concentration in the phase - 0.8×10^{-4} M)

The linear dependence of the capacity parameter (1/k′) for the amino acids on the concentration of the Chemosensor I receptor (Fig. S9) indicates the formation of 1:1 receptor–amino acid complexes in the mobile phase.

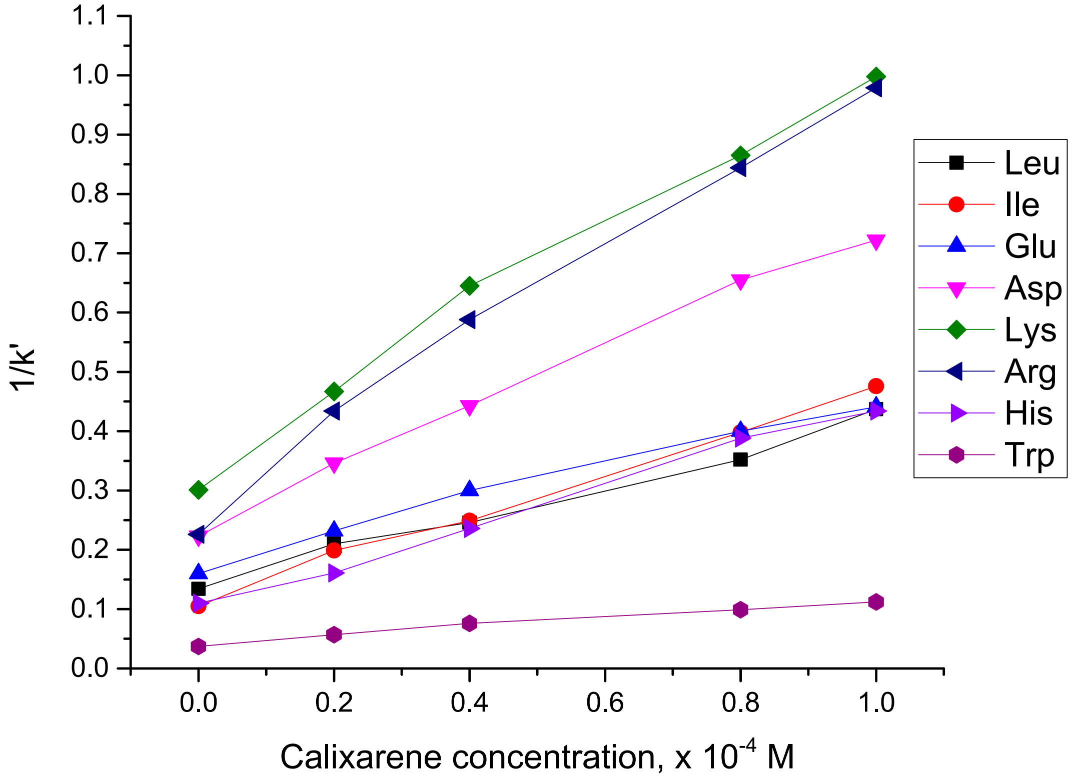

Figure S9. The dependences of (1/*k′*) values of the amino acids on the concentration of Chemosensor I receptor in the mobile phase

The linear dependence allows using equation (1) to calculate the stability constants (K_A) of the host-guest complexes:

$$1/k' = 1/k_0' + K_A \times [CA] / k_0' \quad (1),$$

where: (k_0') and (k') are the capacity factors of the amino acid determined before and after the addition of calixarene to the mobile phase; [CA] is the concentration of the calixarene in the mobile phase.

A detailed description of the determination of (K_A) values is provided in the articles [38,39,51].

Responses of chemosensor I to amino acids

Figure S10 presents the typical responses of Chemosensor I to L-arginine, to which it exhibited the highest sensitivity, L-lysine, to which it showed the second-highest sensitivity, and L-histidine and L-isoleucine, to which it exhibited the lowest sensitivity.

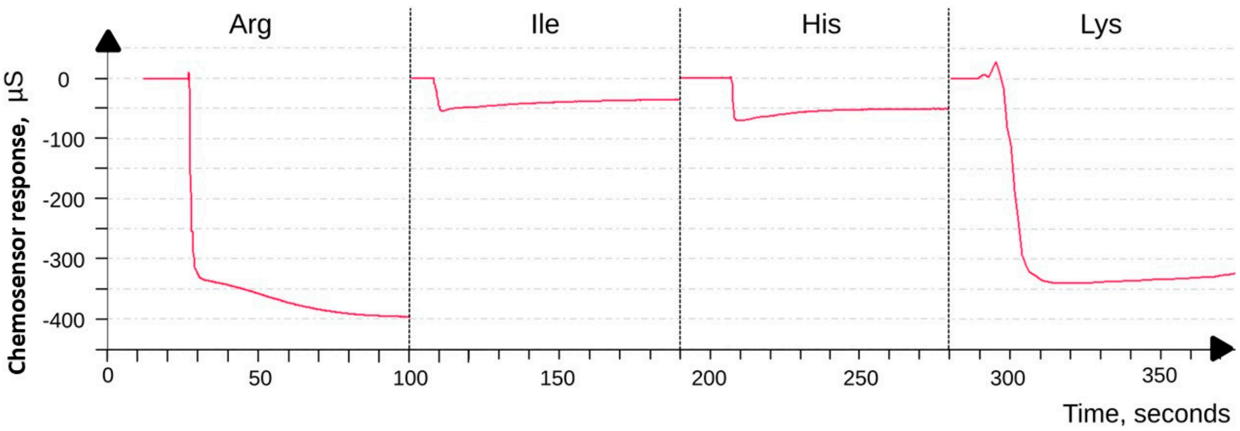

Figure S10. Responses of Chemosensor I to L-arginine, L-isoleucine, L-histidine and L-lysine.
